# Diverse Functions of mRNA Metabolism Factors in Stress Defense and Aging of *Caenorhabditis elegans*

**DOI:** 10.1371/journal.pone.0103365

**Published:** 2014-07-25

**Authors:** Aris Rousakis, Anna Vlanti, Fivos Borbolis, Fani Roumelioti, Marianna Kapetanou, Popi Syntichaki

**Affiliations:** 1 Biomedical Research Foundation of the Academy of Athens, Center of Basic Research II, Athens, Greece; 2 Faculty of Medicine, University of Athens, Athens, Greece; 3 Faculty of Biology, School of Science, University of Athens, Athens, Greece; 4 Department of Biology, School of Science and Engineering, University of Crete, Heraklio, Crete, Greece; BSRC 'Alexander FLEMING', Greece

## Abstract

Processing bodies (PBs) and stress granules (SGs) are related, cytoplasmic RNA-protein complexes that contribute to post-transcriptional gene regulation in all eukaryotic cells. Both structures contain translationally repressed mRNAs and several proteins involved in silencing, stabilization or degradation of mRNAs, especially under environmental stress. Here, we monitored the dynamic formation of PBs and SGs, in somatic cells of adult worms, using fluorescently tagged protein markers of each complex. Both complexes were accumulated in response to various stress conditions, but distinct modes of SG formation were induced, depending on the insult. We also observed an age-dependent accumulation of PBs but not of SGs. We further showed that direct alterations in PB-related genes can influence aging and normal stress responses, beyond their developmental role. In addition, disruption of SG-related genes had diverse effects on development, fertility, lifespan and stress resistance of worms. Our work therefore underlines the important roles of mRNA metabolism factors in several vital cellular processes and provides insight into their diverse functions in a multicellular organism.

## Introduction

A plethora of evidence in all eukaryotes has established that aging is a multifactorial process that can be modulated by several means, ranging from genes to lifestyle and pharmacological interventions. Genetic and genome-wide studies in many organisms shed light on the basic molecular mechanisms of aging and identified conserved signaling pathways as master modulators of lifespan [1]. Fundamental cellular functions that primarily alter metabolism or preserve homeostasis can influence the rate of aging and age-related disability/degeneration. Such cellular functions comprise the activation of stress response and repair mechanisms, the enhancement of catabolic processes and the reduction of anabolic processes. Protein synthesis is an anabolic process that, when reduced by gene mutations, drugs, hormonal or stress signals, increases lifespan in diverse species. The underlying mechanisms could involve energy conservation and/or reprogramming of gene expression that is essential for cell survival [2]. In accordance with this, several studies demonstrate that protein synthesis is tightly regulated by environmental stress in eukaryotes. Protein synthesis rates are mainly controlled at the level of translation initiation but post-transcriptional regulation of mRNAs, including processing, export, decay and localization, can also affect translation. Accordingly, the impact of post-transcriptional regulation of gene expression by RNA-binding proteins and cytoplasmic RNA granules is an emerging field in regulators of aging and age-related diseases [3]–[5].

Processing bodies (PBs) and stress granules (SGs) are major cytoplasmic RNA-protein complexes that regulate, in part, the abundance and translation of mRNAs in all eukaryotic cells. PBs contain non-translatable mRNAs and a variety of proteins involved in translational repression, 5′ to 3′ mRNA decay and RNA-induced silencing [6]–[8]. SGs function in mRNA storage following environmental stress and contain stalled 43S pre-initiation complexes and many RNA-binding proteins that regulate the stability, storage or translation of mRNAs [9]. Both PBs and SGs are highly dynamic structures that share specific components and can spatially interact to determine the fate of mRNAs in the cytoplasm, in equilibrium with translating polysomes [10]–[14]. However, the physiological role of these complexes has not yet been fully elucidated; the processes of translation inhibition and mRNA decay can occur normally in yeast, *Drosophila* and mammalian cells that are defective in PB or SG formation, suggesting that their accumulation is a consequence of rather than a perquisite for mRNA metabolism [15]–[19]. Furthermore, under some conditions in yeast, decapping and 5′ to 3′ decay occur while mRNAs are still associated with polyribosomes [20]. Nevertheless, the evolutionary conservation of both RNA granules supports that they function to impart specificity in molecule interactions and efficiency in translation repression and mRNA degradation.

Both PBs and SGs participate in the transient translational silencing and global reprogramming of gene expression that occur in response to cellular stress [8]. They accumulate within minutes of stress and dissolve within a few hours of stress recovery. The aggregation of non-translatable mRNAs into PBs and SGs could either promote or inhibit mRNA decay and might be crucial for the adaptation or recovery of cells e.g. through the stabilization of specific transcripts. These include either stress-responsive transcripts with short half-lives [21] or energy-costly mRNAs, as those encoding the abundant cytoplasmic ribosomal proteins [22]. In addition, SGs can affect key signaling pathways involved in the global stress response, such the MAPK, TOR and HIF-1 pathways [23]–[26]. Studies in yeast and mammalian cells have shown that stress-induced granules can differ in composition and assembly rules, depending on the type of stress insult [11], [27].

In *C. elegans* several types of RNA granules have been described during the processes of oogenesis and embryogenesis. P granules are a type of germ granule, required for germ cell development, with a primary role in post-transcriptional regulation of maternal mRNAs in the gonad [28]–[31]. Other RNA granules accumulate within the *C. elegans* gonad in response to developmental or environmental signals and differ in composition and function [32]–[34]. All these diverse RNA granules participate in proper mRNA control during early development or in aged/stressed oocytes and share some components with somatic PBs and SGs in other organisms[32]–[37]. Such components include the worm homologues of proteins implicated in mRNA decapping or translational repression, as the decapping enzyme DCAP-2 (Dcp2 in yeast and mammals) and its cofactor DCAP-1 (Dcp1), the translation regulator CGH-1 (Dhh1/RCK), the decapping activators PATR-1 (Pat1) and LSM-1 (Lsm1) or the 5′ to 3′ exonuclease XRN-1 (Xrn1). In addition, they harbor RNA-binding proteins, such as the worm homologues of human ataxin-2 (ATX-2), the poly(A) binding protein PABP (PAB-1) and TIA-1/TIAR proteins (TIAR-1, -2) that are SG components in mammalian cells. Although the structure, localization and function of these germline and early embryonic RNA granules have been thoroughly characterized, somatic PBs and SGs have not yet been well investigated in *C. elegans*. The developmental consequences of loss of several P granule components have been nicely described [34]–[36], [38] but the effect of somatic PB and SG components on survival of the organism during stress conditions or aging are largely unknown.

Here, we present data on worm's stress survival and lifespan modulation by factors involved in mRNA metabolism. We first provide *in vivo* insights into the dynamic formation of somatic PBs and SGs in intact adult animals, in response to various environmental stresses. Moreover, an age-dependent accumulation of PBs, but not SGs, was observed in somatic cells of worms. Aggregation of PBs in young adults is also triggered in response to alterations in 5′ to 3′ mRNA degradation but not to reduced mRNA translation. Furthermore, we show that direct alterations in PB-related genes, by mutations or RNA interference (RNAi), can influence lifespan and stress resistance, beyond their developmental role. We reveal that stress conditions induce distinct modes of SG formation and deletion of SG-related genes has diverse effects on development, lifespan and stress response of worms. Our work provides genetic characteristics of proteins localized to PBs and SGs and offers new insights into the pattern and function of these RNA granules at the organismal level.

## Materials and Methods

### *C. elegans* strains and culture

Standard methods of culturing and handling worms were used [39]. Worms were raised on NGM plates seeded with *Escherichia coli* OP-50 or HT115 (DE3) for RNAi experiments, at the indicated temperature. Wild-type Bristol N2 and some mutant strains were provided by the *Caenorhabditis* Genetics Center (CGC, University of Minnesota), which is funded by NIH Office of Research Infrastacture Programs (P40 OD010440). Other mutant strains were provided by the Mitani Lab through the National Bio-Resource Project of the MEXT, Japan. Table S1 shows all strains used in this study. All single mutants were outcrossed 3–5 times with N2 and relevant mutations were tracked in F2 progeny by PCR (Table S2). Double mutants were made by crossing the relevant strains and PCR-based selection in F2 progeny. Transgenic animals were generated by microinjection of plasmid DNAs into the gonad of N2 young adults, using *rol-6(su1006)* as co-transformation marker [40]. Multiple lines were generated for each genotype and screened for the representative expression pattern. Transgenic mutants were generated by crossing N2 transgenic hermaphrodites with males of the desired mutant background.

### Constructs

RNAi plasmids were constructed by inserting gene-specific PCR product, amplified from genomic DNA using the appropriate primers (Table S2), into the L4440 feeding vector (pPD129.36) [41]. For the double *dcap-1/-2(RNAi)* construct both gene fragments were cloned into a single L4440 vector [42]. See Table S3 for RNAi construct details. The presumptive promoter of *dcap-1* (∼500-kb) was obtained by PCR (Table S2) and inserted into the Fire Lab vector pPD95.77 for the *P_dcap-1_*::*gfp* transcriptional fusion. For the *dcap-1::gfp* transgene, the *gfp* sequence was inserted between the end of *dcap-1* coding region and its 3′ UTR in the plasmid FLAG::*dcap-1*, kindly provided by Dr. Min Han [43]. The *tiar-3::gfp* transgene was constructed by cloning the promoter (1969-bp) and coding region in pPD95.77. For *gfp::tiar-1* and *gfp::tiar-2* a modified version of pPD95.77 was used, allowing insertion of the promoter regions of *tiar-1* (1421-bp) or *tiar-2* (1614-bp) upstream of *gfp* with subsequent in-frame insertion of their corresponding coding regions followed by their own 3′ UTRs (489-bp for *tiar-1* and 680-bp for *tiar-2*). The intermediate constructs with the promoter only sequences fused to *gfp* of pPD95.77 were used as transcriptional reporters. The tagRFP fusions were constructed by replacing the GFP by tagRFP, obtaining the relevant sequence by PCR amplification from pHb9::tagRFP (a gift from Dr. Ivo Lieberam).

### RNA interference

RNAi experiments were carried out by adding synchronized L4s or eggs to plates seeded with HT115(DE3) bacteria that express dsRNA for the indicated gene. HT115 bacteria transformed with the relevant RNAi vectors were grown at 37°C in LB medium with ampicillin (50 µg/ml) and tetracycline (10 µg/ml). On the following day, fresh cultures with ampicillin were induced with 1 mM isopropylb-D-thiogalactopyranoside (IPTG) and seeded on RNAi plates [44]. Bacteria carrying the empty vector (pL4440) and treated likewise were used as control cultures (Control(RNAi)).

### Microscopy

The expression pattern of transgenic worms was monitored by mounting levamizole-treated animals on 3% agarose pads, on glass microscope slides. Animals (∼20 animals per condition in at least three experiments) were imaged using a Leica TCS SP5 confocal imaging system. For imaging assays of transgenic worms at different ages, adults from age-synchronized cultures, raised at the indicated temperatures from hatching, were picked and monitored under the same microscopy settings. For heat-shock assays 1-day adults were shifted at 35°C for the indicated time and were immediately monitored. For recovery assays the heat-shocked animals were incubated for 0.5–3 h at 20°C before mounting. For oxidative stress 1-day adults were transferred on NGM plates seeded with UV-killed OP-50 bacteria containing 10–15 mM sodium arsenite and were visualized after 2–3 h. For osmotic stress 1-day adults were transferred on NGM plates containing 350 mM NaCl and were visualized after 0.5–1 h. All images were taken at 20× magnification, keeping the same microscopy settings for each strain per condition. Images shown from confocal are 2D maximal projections of z-stacks, or optical sections where is indicated, processed in Photoshop CS3.

The quantification of PBs formed under HS was performed as follows: three random squares of 50 µm×50 µm were drawn along a worm; the number of aggregates within each square was measured and their mean value, representing the number of granules per 2500 µm^2^ per worm, was calculated. This procedure was repeated for a total of 20 worms from at least two independent experiments and the average of the mean values is presented in Table S4. The same method was applied to measure SGs visualized using the P*_myo-3_*::GFP::TIAR-2 marker. In the case of age-induced PBs and SGs we measured aggregates formed in the area of the head (from the pharynx to the tip of the worm) to avoid the intestinal autofluorescence. The same method was used to measure TIAR-1 granules as well as SA-induced PBs. When using the GFP::TIAR-2 marker, we measured the number of granules formed in 100 µm length of excretory cell. Again a total of 15–20 worms were counted in each case from at least two independent experiments. All measurements were performed using the count tool in Photoshop CS3. Statistical analysis (unpaired *t*-test) was performed using GraphPad Prism version 5 (GraphPad Softwate, San Diego, California USA).

### RNA isolation and quantitative reverse transcription PCR (qRT-PCR)

Total RNA was prepared from frozen worm pellets, of the indicated genetic backgrounds and developmental stages, using a NucleoSpin RNA XS kit (Macherey-Nagel) and measured by Quant-iT RNA Assay Kit (Invitrogen). Total RNA was reverse transcribed with iScriptTM cDNA Synthesis Kit (Biorad) and quantitative PCR was performed using the SsoFastTM EvaGreen Supermix (BioRad) in the MJ MiniOpticon system (BioRad). The relative amounts of mRNA were determined using the Comparative Ct method for quantification and gene expression data are presented as the fold change relative to control. qRT-PCR was performed in triplicates and each sample was independently normalized to endogenous reference *ama-1*. The mean ± the standard deviation (SD) of at least two independent experiments is presented. All statistical comparisons were performed by Student's *t*-test for unpaired samples in GraphPad Prism 5.The primer sequences for qRT-PCR are available upon request.

### Western Blotting

About 1500–2000 synchronized worms of each strain, grown on OP50 plates at 25°C, were subjected to heat-shock (35°C for 3 h), in the first day of adulthood and were collected in M9 buffer, washed 2–3 to remove bacteria and frozen in ethanol dry ice. Control worms (no heat-shocked) of the same age were treated similarly. Likewise, ∼1500 L4-synchronized worms of each strain at 25°C, were transferred on OP50 plates containing 50 µM 5-fluoro-2'-deoxyuridine (FUDR) to prevent progeny production and collected as either 1-day or 5-day adults in M9 buffer. After 2–3 washes to remove bacteria they were frozen in ethanol dry ice. In all cases, before loading onto SDS-PAGE, worm pellets were boiled in 2× SDS-sample buffer for 10 min. Worm lysates were resolved on a 10% SDS-PAGE, western blotted and analyzed with primary antibodies against to either GFP (1∶10,000, BD Biosciences) or actin (1∶5,000, Clone C4, Millipore). Secondary anti-rabbit and anti-mouse IgG antibodies (HRP) were used respectively for immunoblot signal detection with ECL (Thermo Scientific). Quantification of immunoblot signals was performed using ImageJ software. Ratio of DCAP-1::GFP to actin levels was measured in two independent experiments.

### Lifespan assays

Lifespan analysis was conducted at 20°C or 25°C as described previously [45]. Briefly, eggs or mid-to-late L4 larvae of each strain (at least 70 animals per experiment) were transferred to NGM plates seeded with OP-50 or RNAi bacteria of interest and were moved to fresh plates every two days. Viability of the worms was daily scored and animals that failed to respond to stimulation by touch were referred as dead, whereas that bagged, ruptured or crawled off the plates were referred as censored in the analysis. For post-developmental assays, day 0 of adulthood was defined as the day that mid-to-late L4s were transferred to plates. Lifespan and statistical analysis were performed using GraphPad Prism version 5. Each population is compared with the appropriate control population and p-values were determined using the log-rank (Mantel-Cox) test.

### Fertility assay

Worms of each genotype were grown at 20°C or 25°C and 5–15 L4 hermaphrodites were placed on individual NGM plates to lay eggs. Animals were transferred daily to fresh plates until egg-laying ceased and the hatched progeny were counted in each plate. The total number of progeny per worm (brood size) was counted and the average brood size (mean ± SD) of each strain was plotted. Unpaired *t*-test was used to calculate p-values in GraphPad Prism 5.

### Stress resistance assays

For heat-shock assays, 1-day adult worms were shifted to 35°C for 6 h (8 h for *daf-2* mutants). After 16 h of recovery at 20°C the percentage of worms surviving was determined. For UV resistance assays 5-day adults were irradiated on plates without bacteria at 0.2 J/cm^2^ and then were transferred to plates with food at 20°C. Three days later the percentage of worms surviving was determined. For oxidative stress 1-day adults of each strain were transferred on plates seeded with UV-killed OP-50 containing 5 mM sodium arsenite and the percentage of worms surviving was determined after 24 h (for transgenic worms) or 48 h (non-transgenic worms). For osmotic stress 1-day adults were transferred on NGM plates containing 400 mM NaCl and scored for survival after 24 h. The average (mean ± SD) of at least three independent experiments with ∼100 individuals for each strain per experiment was plotted. Unpaired *t*-test was used to calculate p-values in GraphPad Prism 5.

## Results

### Induction of PBs in *C. elegans* somatic cells by environmental stress

A well-described marker of PBs in all organisms is the decapping subunit Dcp1, which is encoded by the *dcap-1* (Y55F3AM.12.1) gene in worms (http://www.wormbase.org). DCAP-1 protein is a component of P granules in germline and PB-like structures in embryos [32], [36]–[38]. We generated transgenic animals that carry either the transcriptional (*P_dcap-1_::gfp*) or the translational (*P_dcap-1_::dcap-1::gfp::3' UTR^dcap-1^* referred to as *dcap-1::gfp* for simplicity) fluorescent reporter gene to monitor the expression pattern in somatic cells of adult wild-type (N2) worms, under normal growth conditions (Fig. S1A). Worms expressing the *P_dcap-1_::gfp* reporter (line BRF154, Fig. S1A) displayed a smooth and diffused fluorescent signal throughout the cytoplasm of most cells. Worms bearing the *dcap-1::gfp* fusion (BRF155, Fig. S1A) showed a similar ubiquitous expression pattern with occasional cytoplasmic puncta, varying among the worms. Although several protein components of PBs have an intrinsic capacity to aggregate [16], [46]–[48], in mammalian cell lines PB components show a diffuse distribution in the cytoplasm and only few PBs are formed under normal conditions [49]. Also, high expression levels of fluorescent PB proteins in mammalian or yeast cells can further induce the aggregation of PBs and can alter the cellular stoichiometry of other PB components leading to aberrant structures [48]–[52]. Having established that the *dcap-1::gfp* fusion is functional (see text below and Fig. S5C), we showed by quantitative RT-PCR that the expression levels of *dcap-1* were not higher but underrepresented in BRF155 transgenic animals compared to the physiological levels of *dcap-1* in N2 (Fig. S1B). We interpreted that the transgene silencing effects resulted from the presence of vector backbone, as microinjection of the *dcap-1::gfp* reporter construct in linear, vector-free form [53] greatly improved its expression; a representative transgenic line (BRF261, Fig. S1A) had increased *dcap-1(mRNA)* levels (Fig. S1B) and more punctate pattern under normal conditions. Moreover, in a germline-deficient background (*glp-1*) the *dcap-1::gfp* transgene silencing was abolished (BRF219, Fig. S1A, B) confirming that such effects originate from the germline [54].

**Figure 1:**
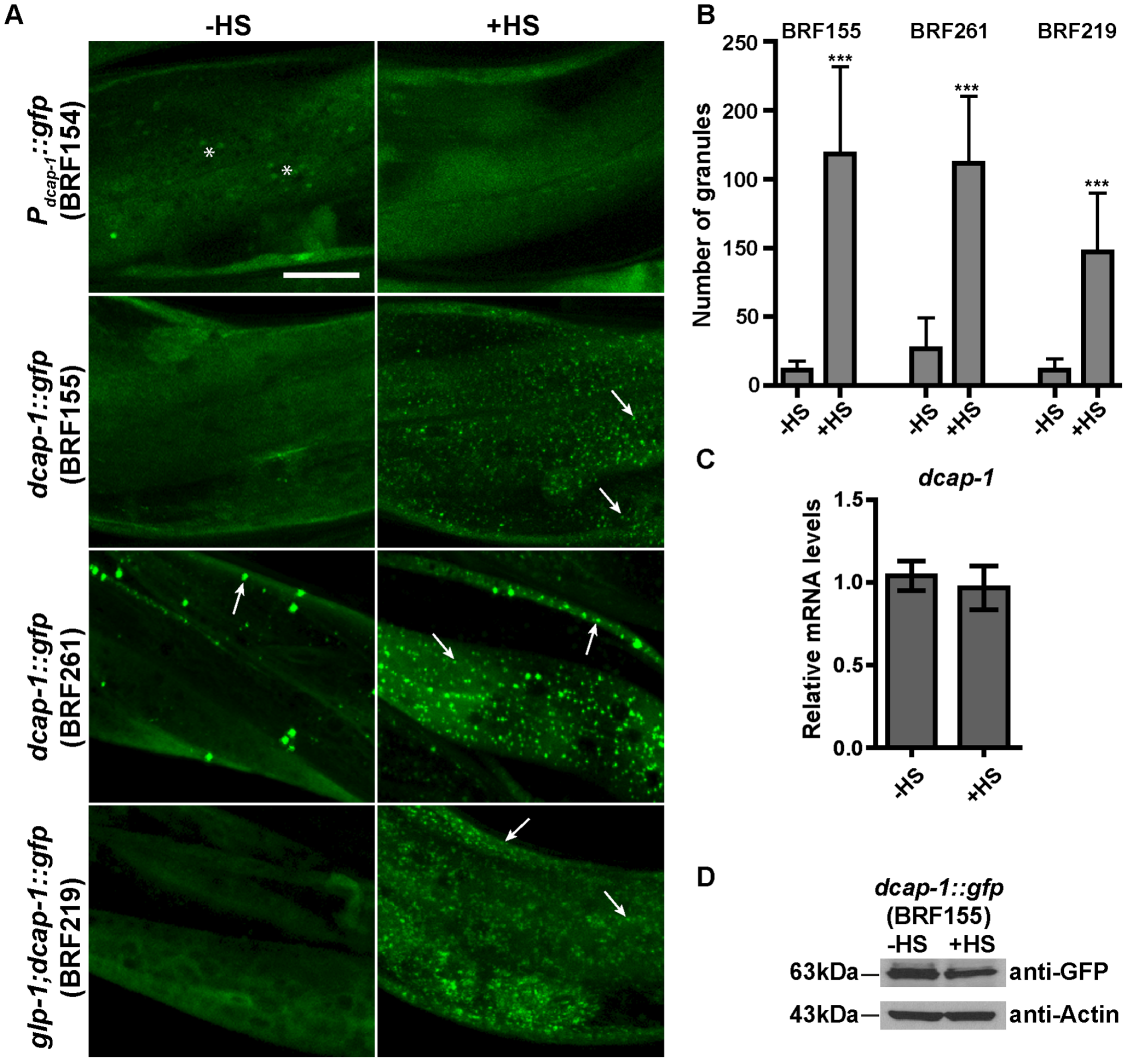
Aggregation of DCAP-1 protein in response to heat-shock. (A) Representative confocal images of somatic tissues in 1-day adult worms expressing the transcriptional fusion *P_dcap-1_*::*gfp* (BRF154) or the translational fusion *dcap-1::gfp* in N2 (BRF155 and BRF261) and germline-deficient *glp-1(e2141)* worms (BRF219), normally grown at 25°C (-HS) or transiently subjected to heat-shock (+HS, 35°C for 3 h). Arrows point to DCAP-1::GFP granules in body wall muscles that are highly induced upon stress. Asterisks denote the intestinal autofluorescence. Scale bar: 25 µm. (B) Quantification of data in (A). Values on Y axis show the number of granules per 2500 µm^2^ per worm (mean±SD, see Materials and Methods and Table S4). (C) Endogenous *dcap-1*(mRNA) levels in 1-day adults N2, grown at 25°C before (-HS) or after heat shock (+HS, 35°C for 3 h) were measured by quantitative RT-PCR and normalized to endogenous *ama-1(mRNA)* levels. Error bars represent the standard deviation of the means of five independent experiments (p-value = 0.3466, calculated by Student's *t*-test). (D) Western blot analysis of DCAP-1::GFP protein expression in 1-day adult BRF155 transgenic animals, before (-HS) or after heat shock (+HS), using anti-GFP or anti-Actin (as a loading control) antibodies. Band intensity of DCAP-1::GFP normalized to actin gives a value of 0.83, showing similar protein levels in the two conditions.

**Figure 2:**
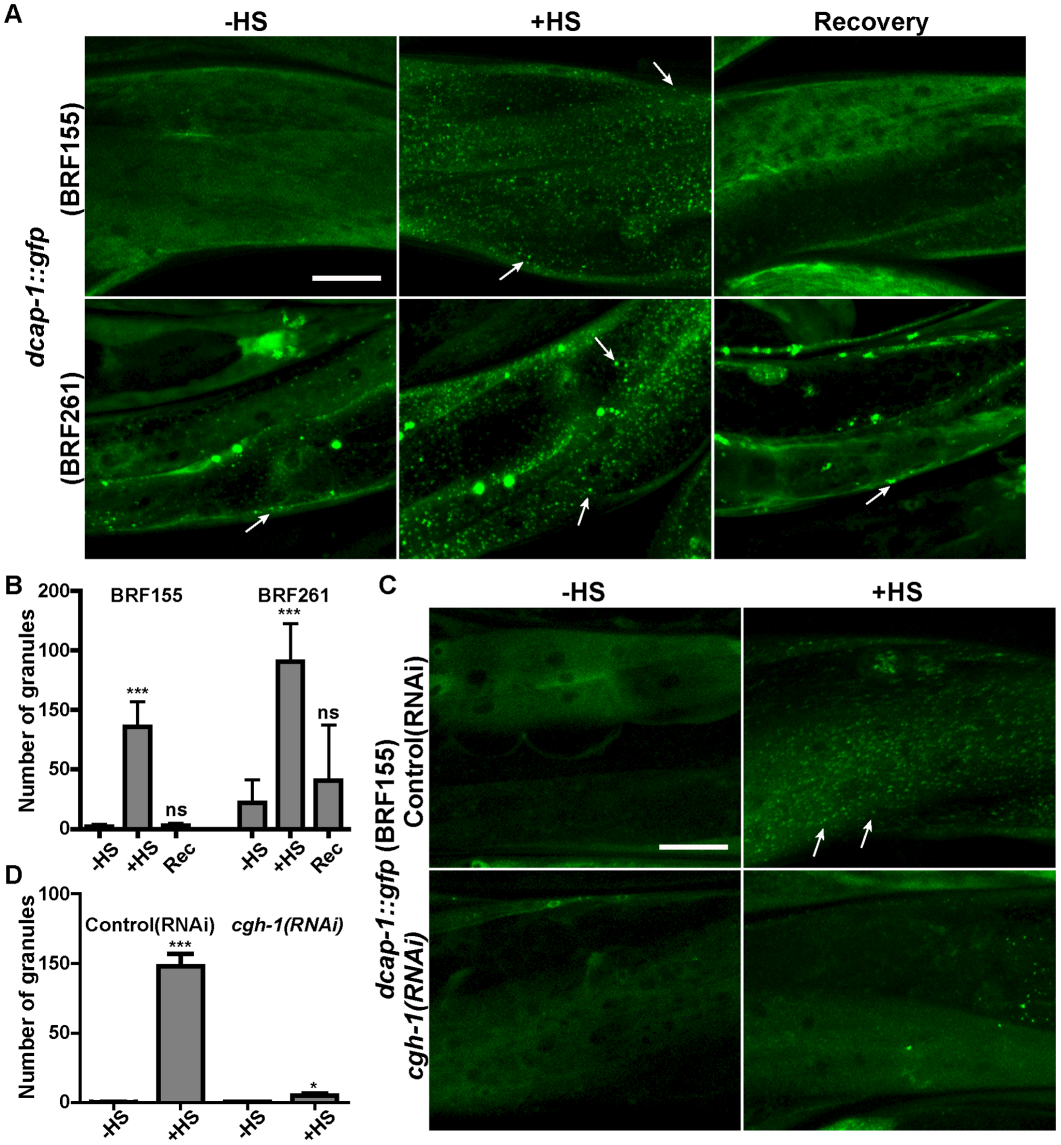
Accumulation of DCAP-1-containing granules under heat-shock is rapid, reversible and sensitive to *cgh-1(RNAi)*. (A) Representative confocal images of 1-day adult worms expressing *dcap-1::gfp* (BRF155 and BRF261 strains) normally grown at 25°C (-HS) or transiently subjected to heat-shock (+HS, 35°C for 3 h),following recovery for 3 h at 20°C (Recovery). (B) Quantification of data in (A). (C) Representative confocal images of 1-day adult worms expressing *dcap-1::gfp* (BRF155) fed from eggs with Control(RNAi) or *cgh-1(RNAi)* bacteria and grown at 25°C (-HS) or subjected to heat-shock at 35°C for 3 h (+HS). In all cases arrows point to DCAP-1::GFP granules in body wall muscles. (D). Quantification of data in (C). Values on Y axis show the number of granules per 2500 µm^2^ per worm (mean±SD, see Materials and Methods and Table S4). Scale bar: 25 µm.

To validate that the DCAP-1::GFP-containing granules represent dynamic PBs in living animals we subjected all the above transgenic worms to heat-shock (+HS, Fig. 1A), a stress that generally enhances PB formation in other organisms, in a reversible manner [51], [55]. Monitoring worms under confocal microscopy immediately after incubation at 35°C for 3 h, resulted in increased number and size of DCAP-1::GFP-containing cytoplasmic puncta in all transgenic lines (Fig. 1A, B). In contrast, there was no change in the expression pattern of worms carrying the transcriptional fusion transgene (BRF154, Fig. 1A), suggesting that the DCAP-1::GFP granules result from the accumulation of diffuse cytoplasmic protein rather than transcriptional or translational induction. This was verified by qRT-PCR for *dcap-1(mRNA)* levels and western blotting for DCAP-1::GFP protein levels, under normal or heat-shock conditions (Fig. 1C, D). The formation of DCAP-1::GFP aggregates in response to heat-shock was also reversible, with the majority of puncta dissolving within 3 h of recovery at 20°C (Fig. 2A, B). Similar enhanced formation of DCAP-1::GFP granules was observed in response to oxidative stress induced by sodium arsenite (BRF155, Fig. S2A). We further showed that RNAi-mediated knockdown of a core PB component, *cgh-1* (encoding a conserved DEAD-box RNA helicase) was sufficient to disrupt the formation of DCAP-1::GFP granules during heat (Fig. 2C, D) or oxidative stress (data not shown), in agreement with studies in yeast or human cells [8], [56].

Another putative component of mammalian PBs is the mRNA-binding protein GW182 [57], which has two homologues in *C. elegans*, AIN-1 and AIN-2, both involved in miRNA-induced silencing function [58]. However, only AIN-1 interacts with components of the decapping complex and co-localizes with DCAP-1 in PB-like structures in *C. elegans*, under normal conditions [43]. Transgenic worms expressing the *ain-1::gfp* reporter have sporadic cytoplasmic puncta under normal conditions, which are further increased after heat-shock (Fig. S2B) without changes in *ain-1(mRNA)* levels (Fig. S2D), similar to *dcap-1::gfp*-expressing animals. We also tested whether the somatic PBs contain the translation initiation factor eIF4E, which localizes to PBs in unstressed mammalian cells but is found to both PBs and SGs upon exposure to stress [12], [56]. In *C. elegans*, *ife-2* encodes the major somatic isoform of eIF4E [59]. Transgenic animals expressing the transcriptional fusion *P_ife-2_::gfp* (BRF68, Fig. S2B) displayed a uniform fluorescence in all cells and did not show any aggregation under heat stress. In contrast, animals carrying the translational fusion *ife-2::gfp*, that drives expression of *ife-2* by its own promoter, had a similar diffused fluorescence pattern under normal growth conditions but rapidly formed numerous cytoplasmic granules in response to heat-shock (Fig. S2B) or oxidative stress (Fig. S2A). In co-localization experiments we monitored that several of heat-induced IFE-2::GFP granules also contain the PB marker DCAP-1::tagRFP (Fig. S2C). However, this partial co-localization could result from docking between SGs and PBs under stress, as it was shown to occur in yeast and mammalian cells [12], [15], [60]. The homogenous cytoplasmic fluorescence pattern of the strong *ife-2::gfp* reporter in unstressed animals argues that IFE-2 does not localize to constitutive PBs. Additionally, we found that *cgh-1(RNAi)*, which prevents stress-induced aggregation of PBs, had no significant effect on the formation and the relative number of IFE-2::GFP granules under heat-shock (Fig. S3A). This indicates that IFE-2 localizes mainly in SGs under stress conditions (see below Fig. S7A). We also found that deletion of *ife-2* did not affect the heat-induced accumulation of PBs (Fig. S3B). Thus, stress-induced PBs in the soma of intact adult worms resemble yeast and mammalian PBs in their pattern, formation and reversibility, albeit differ in localization of IFE-2/eIF4E factor.

### Accumulation of somatic PBs with age

**Figure 3:**
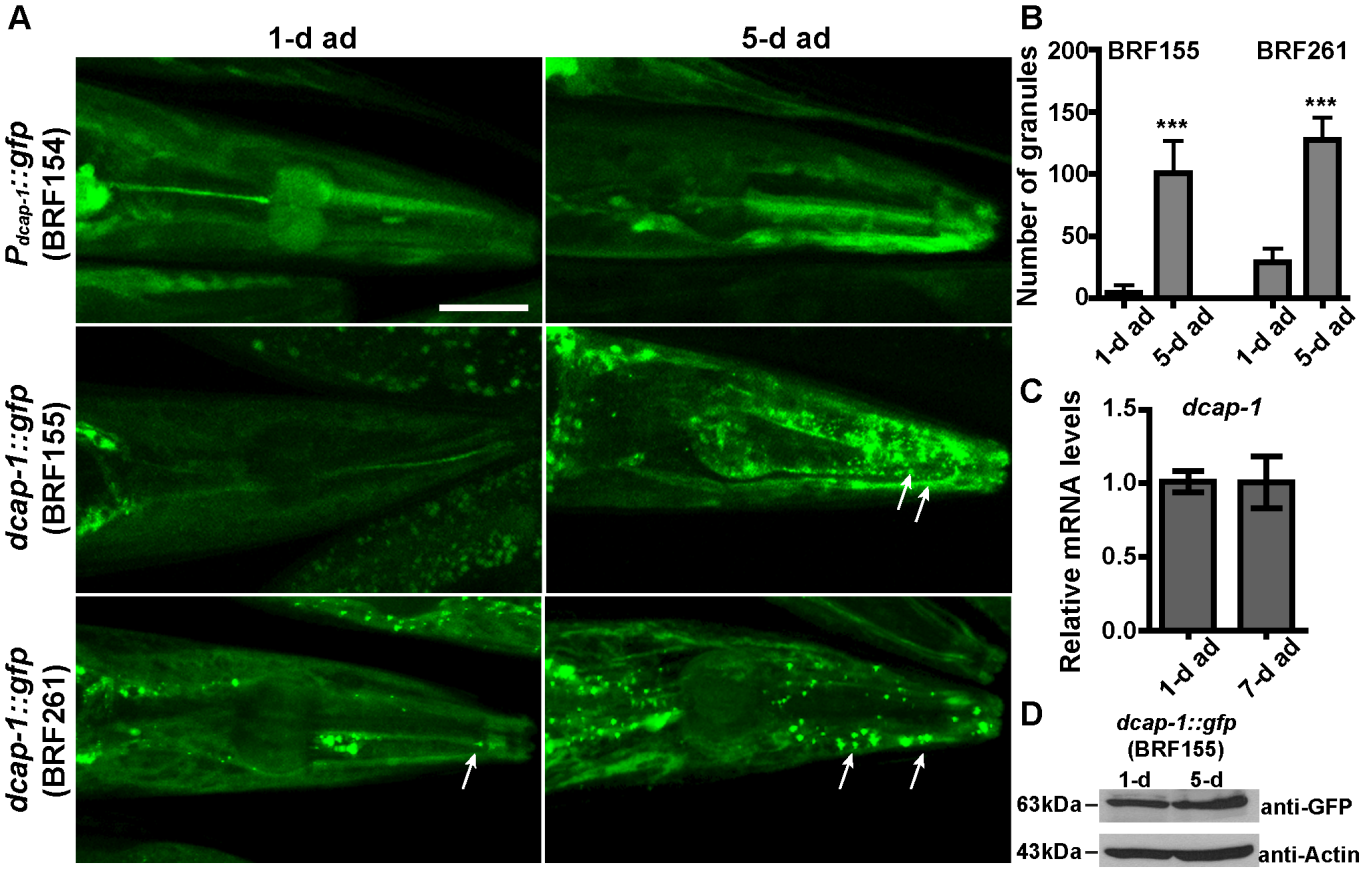
Accumulation of PBs with age. (A) Representative confocal images of 1-day and 5-day adults, grown at 25°C and expressing the transcriptional fusion *P_dcap-1_*::*gfp* reporter (BRF154) or the translational fusions *dcap-1::gfp* (BRF155 and BRF261). Arrows point to age-induced DCAP-1::GFP granules. Scale bar: 25 µm. (B) Quantification of data in (A). Values on Y axis show the number of granules per head (mean±SD, see Materials and Methods and Table S4). (C) Endogenous *dcap-1*(mRNA) levels in 1-day and 7-day N2 adults grown at 25°C before (-HS) or after heat shock (+HS, 35°C for 3 h) were measured by quantitative RT-PCR and normalized to endogenous *ama-1(mRNA)* levels. Error bars represent the standard deviation of the means of six independent experiments (p-value = 0.9697, calculated by Student's *t*-test). (D) Western blot analysis of DCAP-1::GFP protein expression in 1-day and 5-day adult BRF155 transgenic animals, grown at 25°C, using anti-GFP or anti-β-Actin (as a loading control) antibodies. Band intensity of DCAP-1::GFP normalized to actin gives a value of 0.95, showing similar protein levels in the two conditions.

We next explored the pattern of PBs during the aging process, which is associated with marked alterations in protein synthesis and homeostasis [61], [62]. Using the *dcap-1::gfp* reporter we demonstrated that the number and size of PBs gradually increased with age as, there was an increased punctate pattern in 5-day adults compared to 1-day adults, at 25°C (BRF155 and BRF261, Fig. 3A, B). This was not due to altered mRNA or protein levels with age, as verified by qRT-PCR for the endogenous *dcap-1(mRNA)* levels and western blotting for DCAP-1::GFP protein (Fig. 3C, D). The aggregation of PBs with age was also evident in worms grown at 20°C (Fig. S4A, B), albeit in later time-points compared to 25°C consistent with the slower aging process at lower temperatures. Similar age-dependent accumulation of PBs, was reported in worms expressing the DCAP-1::RFP fusion protein [63]. Cytoplasmic granules containing the AIN-1::GFP protein (MH2704, Fig. S2B) also accumulated in aged animals, without changes in *ain-1(mRNA)* levels (Fig. S2D), suggesting that aging can alter the profile of somatic PBs. Interestingly, we did not observe any age-dependent accumulation of the IFE-2::GFP fusion protein (BRF70, Fig. S2B), indicating that IFE-2/eIF4E factor is not part of the PB foci that accumulate with age. Taken together our observations led us to consider that the age-dependent formation of PBs in adult worms could not result from oxidative stress that takes place in aged cells and tissues [64]; this should have induced the assembly of SGs as well, something that we did not monitor by IFE-2 or other SG markers (see below).

**Figure 4:**
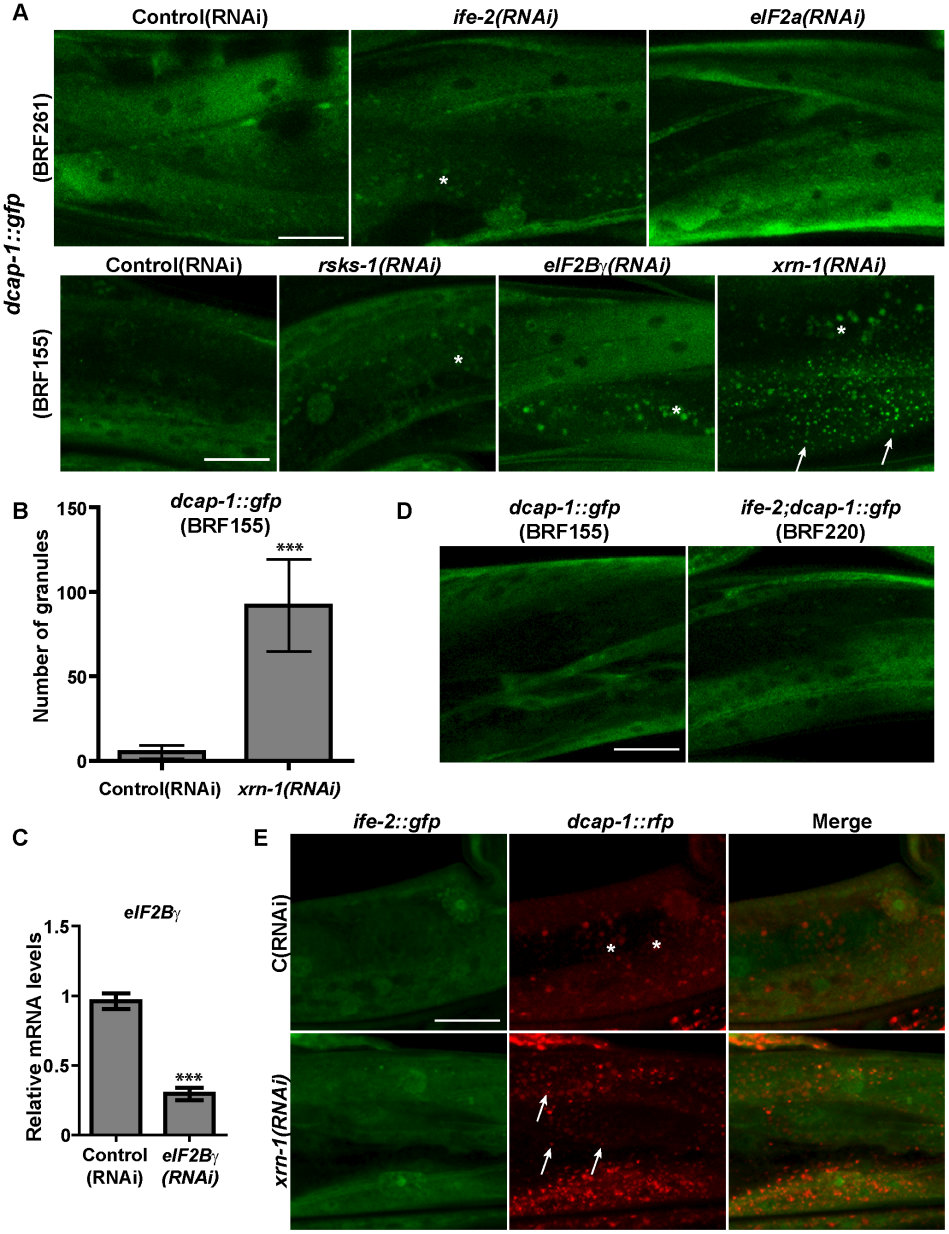
Disruption of mRNA degradation, but not of translation factors, triggers PBs formation that lack *ife-2*/eIF4E. (A) Representative confocal images of 1-day adults expressing *dcap-1::gfp* in N2 (BRF155 and BRF261) fed with *ife-2(RNAi)*, *eIF2a(RNAi), rsks-1(RNAi), eIF2Bγ(RNAi)* or *xrn-1(RNAi)* bacteria. (B) Quantification of data in (A). Values on Y axis show the number of granules per 2500 µm^2^ per worm (mean±SD, see Materials and Methods and Table S4). (C) Endogenous *eIF2Bγ*(mRNA) levels in 1-day adults BRF155 worms grown at 25°C and fed with *eIF2Bγ(RNAi)* from eggs, were measured by quantitative RT-PCR and normalized to *ama-1(mRNA)* levels. Error bars represent the standard deviation of the means of three independent experiments (***p-value<0.0001, in unpaired *t*-test). (D) Representative confocal images of 1-day adults expressing *dcap-1::gfp* in N2 (BRF155) or *ife-2(ok306)* (BRF220) background. (E) Representative confocal images of 1-day adults co-expressing *dcap-1::rfp* and *ife-2::gfp* (BRF313), and treated with *xrn-1(RNAi)* to monitor the lack of co-localization of the two proteins. In all cases, targeted RNAi was initiated from eggs at 25°C in parallel with Control(RNAi) to assess the localization of DCAP-1::GFP into PBs. Asterisks show the intestinal autofluorescence. Scale bar: 25 µm.

We next considered the possibility that reduction of mRNA translation or degradation rates with age might cause or contribute to the accumulation of PBs in aged worms. Studies in yeast and mammalian cells have described the induction of PBs when either translation initiation or mRNA decay is inhibited, due to accumulation of non-translatable mRNAs into PBs [12], [14]. Thus, we performed early in life RNAi of *ife-2* or other translation factors such as eIF2α (encoded by Y37E3.19), *ppp-1*/eIF2Bγ and *rsks-1/*S6K in N2 worms carrying the *dcap-1::gfp* transgene but we didn't observe any PB aggregation, in 1-day adults (Fig. 4A). We validated the efficiency of RNAi clones by qRT-PCR (Fig. 4C) or by testing their effects in the treated adults and their progeny [45], [65]. Consistent with *ife-2(RNAi)*, a strain carrying a null *ife-2(ok306)* mutation and exhibiting lower protein synthesis rates than N2 [45], [65], [66] did not form PBs in adult somatic tissues (*ife-2(ok306); dcap-1::gfp,* Fig. 4D). On the other hand, RNAi of *xrn-1*, the major cytoplasmic 5′ to 3′ exonuclease, resulted in the accumulation of DCAP-1::GFP granules under the same conditions (Fig. 4A, B). Thus, the dynamics of PBs appear to be more prone to regulation by alterations in mRNA degradation rather than in translation rates. We suggest that an age-dependent decline in mRNA decay process could be a causal factor in the onset of PBs aggregation in aged tissues but further experimental validation is needed. Interestingly, *xrn-1(RNAi)* did not cause aggregation of IFE-2::GFP, which remained cytoplasmic in worms that co-express DCAP-1::tagRFP and form distinct granules (Fig. 4E), showing that IFE-2 does not relocalize to PBs under impaired mRNA degradation conditions.

### Disruption of PB-related genes affects development, lifespan and stress response of worms

**Figure 5:**
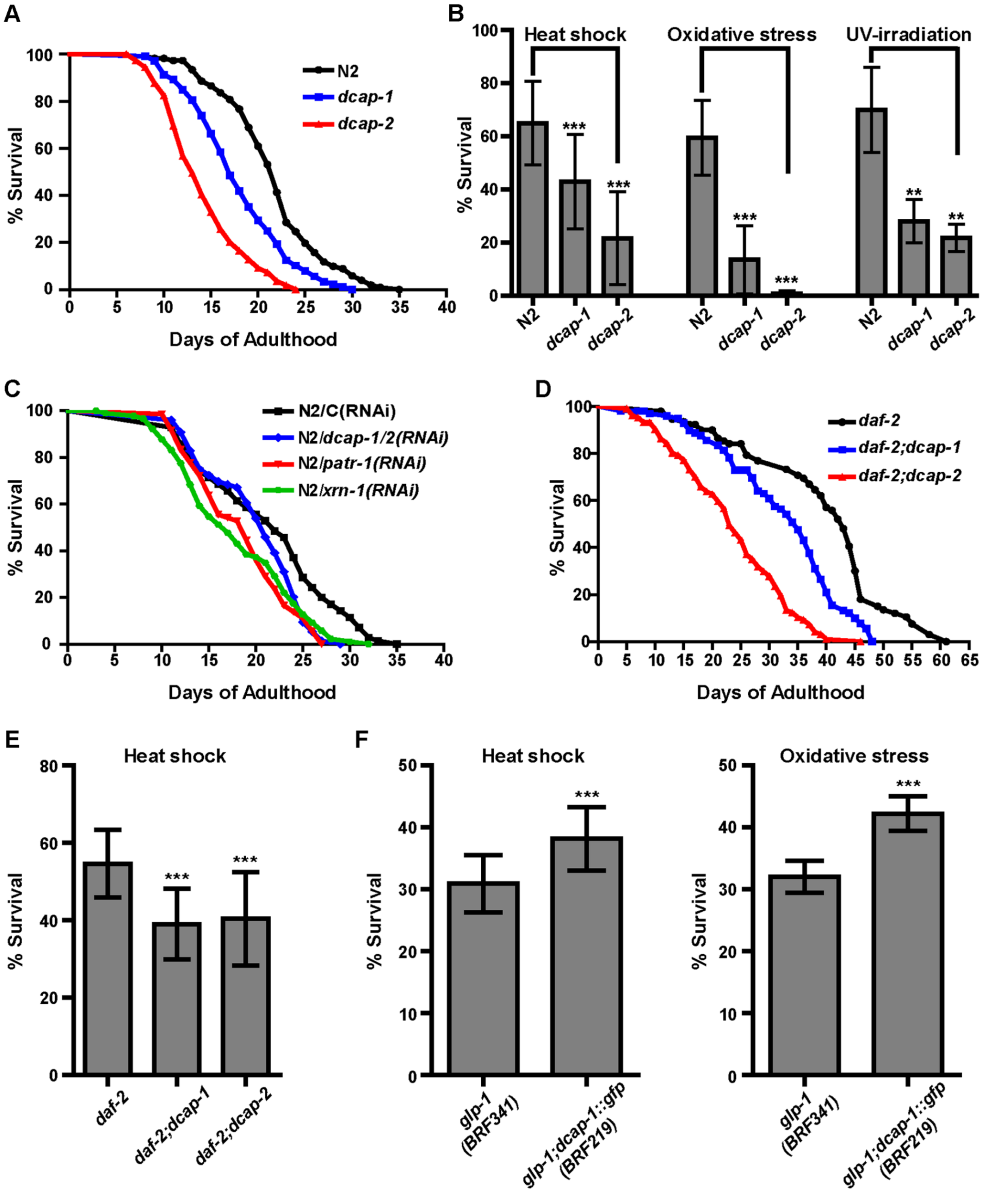
PB components are important for normal lifespan and stress response. (A) Survival curves of N2, *dcap-1* and *dcap-2* mutants at 20°C. (B) Survival of adults *dcap-1* and *dcap-2* mutants in heat-shock (1-day adults at 35°C for 6 h), oxidative stress (1-day adults in 5 mM sodium arsenite for 48 h) and UV-irradiation (5-day adults at 0.2 J/cm^2^), compared to N2. (C) Survival curves of N2 worms treated post-developmentally with *dcap-1/-2(RNAi)*, *patr-1(RNAi)* or *xrn-1(RNAi)* at 20°C. (D) Lifespan of *daf-2*, *daf-2; dcap-1* and *daf-2; dcap-*2 mutants at 20°C and (E) survival of 1-day adults after heat-shock (35°C for 8 h). (F) Survival of 1-day adult *glp-1* germline-deficient mutants that overexpress *dcap-1::gfp* after heat-shock (at 35°C for 6 h) or oxidative stress (5 mM sodium arsenite for 48 h). In lifespan assays the p-values were determined using the log-rank test (see Tables S5 and S6) and in stress assays the error bars show the SD in unpaired *t*-tests (see Materials and Methods). ** indicates very significant (p-value 0.001 to 0.01); *** indicates extremely significant (p<0.001).

We further set out to evaluate the importance of PB-related genes in the physiology of worms and whether disruption of their function could protect from or contribute to mortality. Worms bearing mutations in the decapping genes *dcap-1(tm3163)* and *dcap-2*(*ok2023)* showed a profound delay in development (Fig. S5A) and reduced brood size (Fig. S5B) compared to N2, at both growth temperatures (20 and 25°C). They also exhibited high levels (∼50%) of matricidal death (“bagging” due to internal hatching of eggs) and uncoordinated locomotion. Both mutants had a significantly shorter lifespan than N2 (Fig. 5A and Table S5) and were more sensitive, as adults, in a range of environmental stressors (Fig. 5B). When we expressed the *dcap-1::gfp* reporter in *dcap-1(tm3163)* we rescued the growth and lifespan defects and restored resistance to stresses (Table S5 and Fig. S5C). We noticed that the mutant phenotypes of *dcap-1* were more severe at 25°C compared to 20°C due to a possible residual function of the mutated DCAP-1 protein; *dcap-1(tm3163)* harbors a 334-bp deletion that results in a truncated mRNA, generating a new amino-acid sequence at position 263 (E263) of the C′-terminus of wild-type protein (Fig. S5D). Since the C′ end of DCAP-1 contains a conserved trimerization domain, which in metazoan is required for incorporation of Dcp1 into PBs and mRNA decapping *in vivo* [67], it is possible that the allele *tm3163* doesn't completely remove gene function at 20°C, but at higher temperature (25°C) its phenotypes are greatly enhanced. Therefore, the lifespan of *dcap-1* worms is ∼25% shorter than N2 at 20°C and ∼45% shorter than N2 at 25°C, resembling the lifespan of the null *dcap-*2 mutants (Table S5). Also, each gene disruption by RNAi from the time of egg hatching resulted in similarly decreased lifespan of N2 (Table S6).

We considered that the mortality of decapping mutants could emerge from their early developmental defects. To assess the effects of PB components on lifespan beyond development we performed RNAi post-developmentally, by initiating RNAi during late larval stages of N2. Although the effect of single decapping gene depletion was small we observed significantly decreased lifespan when *dcap-1* and *dcap-2* were simultaneously inactivated *(dcap-1/2(RNAi)* compared to Control(RNAi)-treated worms (Fig. 5C and Table S6). This is consistent with the model of a more efficient disruption of decapping activity when both decapping subunit genes are disrupted [36]. Similarly, post-developmental depletion of *xrn-1*, *patr-1* or *cgh-1* significantly shortened lifespan of N2 (Fig. 5C and Table S6). Given that *xrn-1(RNAi)* induced the accumulation of PBs we postulate that this accumulation is not sufficient to confer longevity, probably due to impaired degradation of resident mRNAs. Furthermore, disruption of *dcap-1* or *dcap-2* had an impact in the lifespan of several long-lived mutants. Post-developmental RNAi-treatment of worms lacking the insulin/IGF-1-like receptor *daf-2* gene [68], [69] resulted in shortening of their extreme long life (Table S6). Similarly, RNAi of decapping genes during adulthood in *ife-2(ok306)* or *rsks-1(ok1255)* translation-defective worms [45], in dietary-restricted *eat-2(ad465)* animals [70] as well as in somatic tissues of the germline-deficient mutant *glp-1(e2141)* [71], reduced their longevity (Table S6). Likewise, introducing the *dcap-1(tm3163)* or *dcap-2*(*ok2023*) alleles in the genome of these long-lived mutants resulted in significantly reduced lifespan and stress resistance compared to controls (Fig. 5D, E for *daf-2* and Table S5), suggesting that the decapping mutant phenotypes were not rescued by any longevity pathway. On the other hand, overexpression of *dcap-1* in somatic tissues of *glp-1* mutants had a positive effect on their ability to cope with stress during adulthood, as it significantly enhanced survival under heat and oxidative stress (Fig. 5F). Taken together, our data indicate that genes directly related to PBs modulate adult lifespan, separately from their developmental role and alterations in functions of PB components increase the mortality rate and the sensitivity of adult worms to various stressors.

### Accumulation of SGs in somatic cells upon stress but not with age

**Figure 6:**
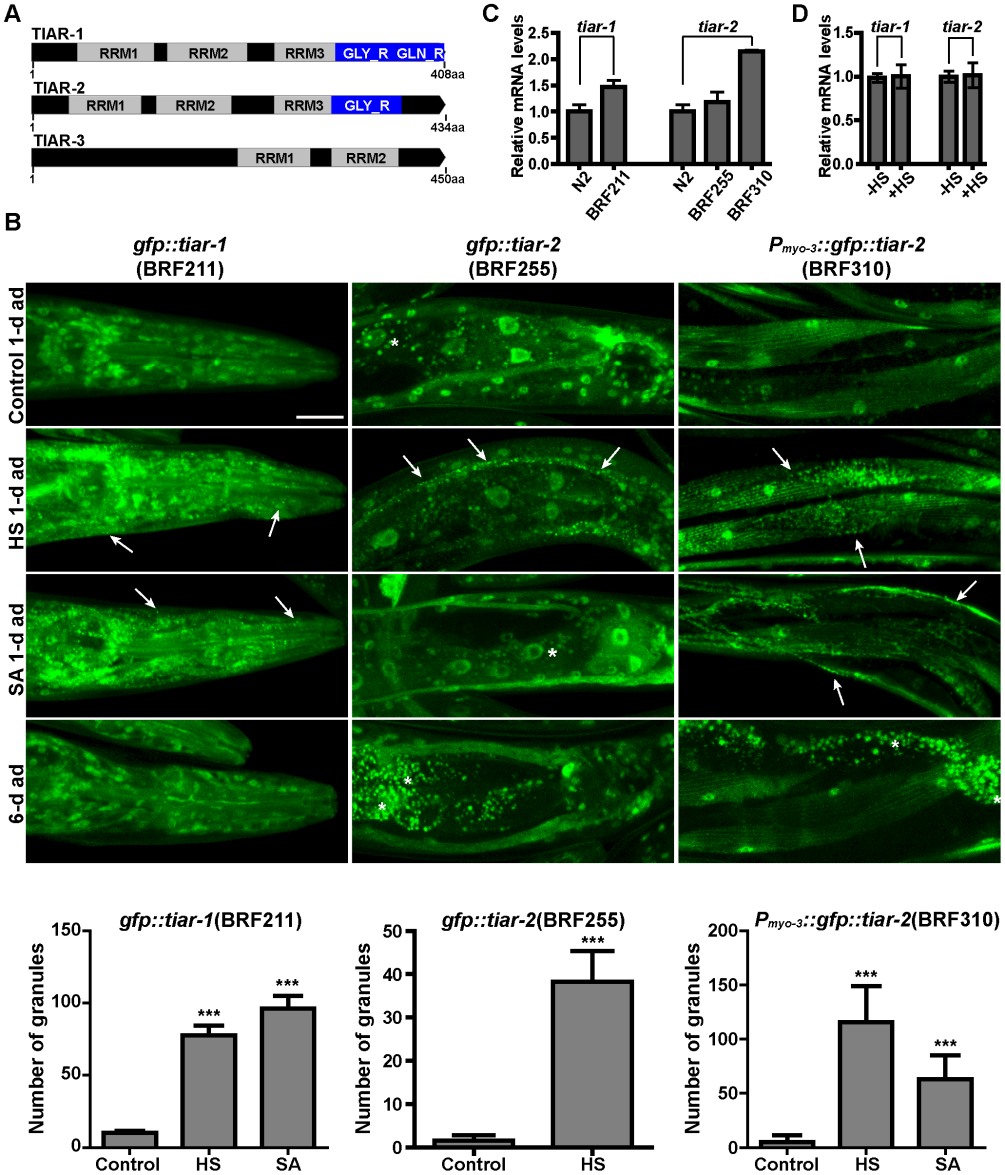
Aggregation of TIAR-1 and TIAR-2 proteins in response to stress but not to age. (A) The domain structure of TIAR proteins, designed using the Prosite MyDomains (http://prosite.expasy.org/mydomains/). The graphic was created using the Exon-Intron Graphic Maker (http://wormweb.org/exonintron). RRM: RNA-recognition motif, GLY_R: Glycine rich domain, GLN_R: Glutamine rich domain. (B) Representative confocal images of 1-day adults of the indicated transgenic strains grown at 25°C (Control 1-d ad), upon heat-shock (HS, 35°C for 3 h), oxidative stress (SA, 15 mM sodium arsenite for 3 h) or compared to 6-day adults under normal conditions. Arrows point to formed granules and quantification of the granule number is shown in the lower panel for each strain. Values on Y axis show the number of granules per head (BRF211), per 100 µM length of excretory cell (BRF255) or per 2500 µm^2^ per worm (mean±SD, see Materials and Methods and Table S4). Asterisks show the intestinal auto-fluorescence. Scale bar: 25 µm. (C–D) Expression levels of *tiar-1* or *tiar-2* genes in (C) the indicated transgenic strains expressing *gfp::tiar-1* and *gfp::tiar-2* by their own promoter (BRF211 and BRF255, respectively) or the muscle specific *P_myo-3_::gfp::tiar-2* (BRF310) and (D) N2 worms after heat shock (35°C for 3 h). Quantification of each mRNA level, relative to *ama-1(mRNA)* levels, in 1-day adults and the mean ± SD of biological triplicates are shown (p>0.05 in Student's *t*-tests).

PBs are functionally related to SGs that have a crucial role in cellular response to environmental stress. SG components in human cells include TIA-1 (T-cell intracellular antigen-1) and TIAR (TIA-1-related) RNA-binding proteins that regulate mRNA stability, in addition to other cellular functions [72]. In *C. elegans* genome (http://www.wormbase.org), three genes, named *tiar-1* (C18A3.5), *tiar-2* (Y46G5A.13) and *tiar-3* (C07A4.1) encode homologues of human TIA-1/TIAR family proteins. The predicted proteins TIAR-1 and TIAR-2 contain three RNA-recognition motifs (RRMs) at their N′-termini and prion-related domains at their C′-termini, whereas TIAR-3 has only two RRMs and no prion-related domain (Fig. 6A). The latter domain in human TIA-1/TIAR proteins is essential for the assembly of SGs under stress [73]. We generated animals expressing transcriptional or translational *gfp*-reporters of *tiar* genes and through observation of several transgenic lines we concluded that: (a) the promoter of both *tiar-1* and *tiar-2* is expressed in most, if not all, cells (*P_tiar-1_::gfp* and *P_tiar-2_::gfp* in Fig. S6); (b) the *gfp::tiar-1* and *gfp::tiar-2* N′-terminal fusion genes, driven by their own promoters, display a diffused fluorescent signal dispersed in both nucleus and cytoplasm (Fig. S6); (c) *tiar-3::gfp*, a C′-terminal fusion driven by its own promoter, is exclusively nuclear, strongly expressed mainly in spermatheca, ventral nerve cord and some head and tail neurons (Fig. S6). We could not detect expression of GFP fusions in germline due to silencing of transgenes in this tissue [74] but previous studies have shown the expression of TIAR-1 [33], [75] and TIAR-2 [32] in germ granules.

**Figure 7:**
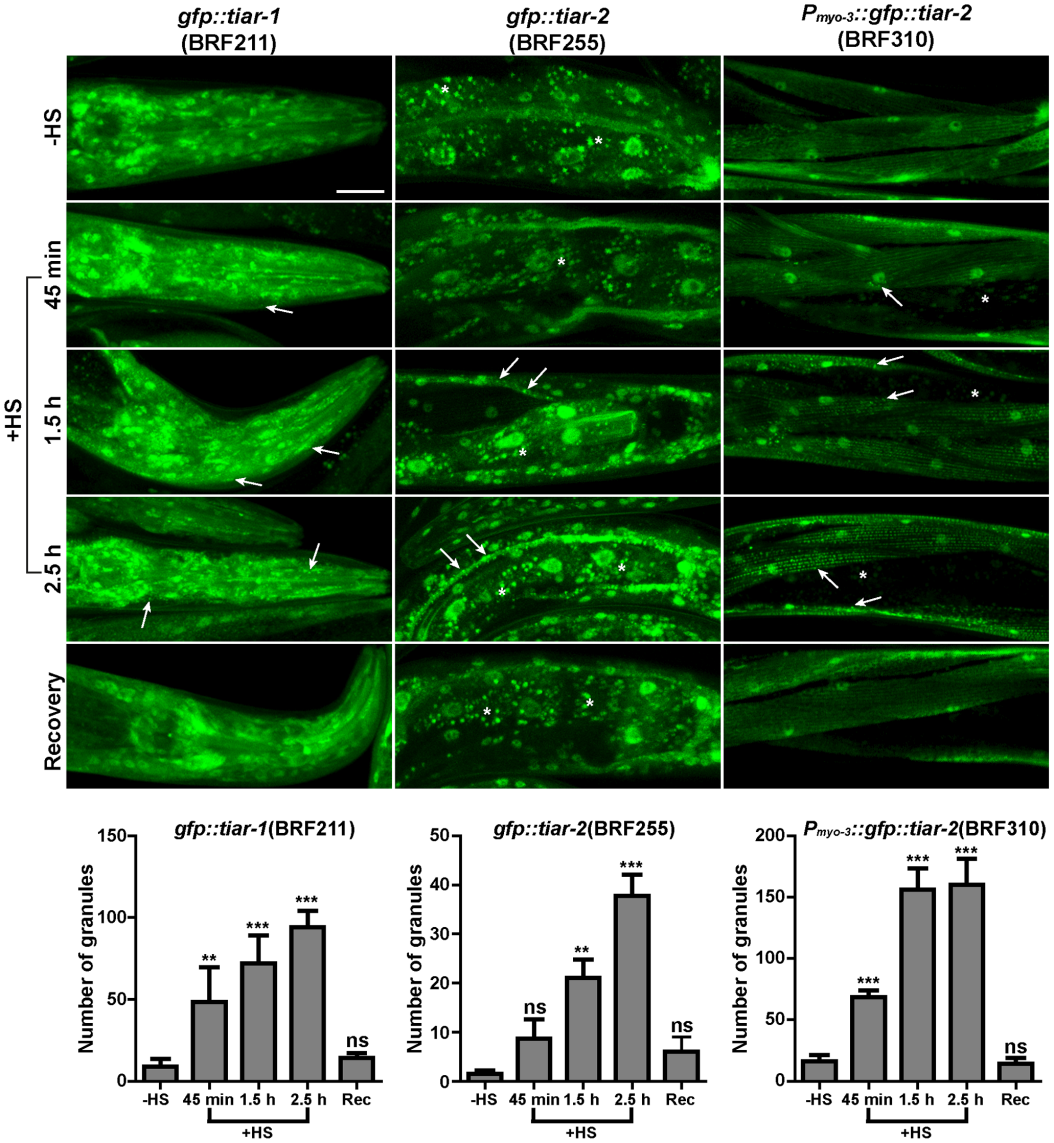
Rapid formation and dissociation of heat-induced SGs. Representative confocal images of 1-day adults expressing *gfp::tiar-1* and *gfp::tiar-2* by their own promoter (BRF211 and BRF255, respectively) or the muscle specific *P_myo-3_::gfp::tiar-2* (BRF310) under normal growth conditions (-HS) and upon heat-shock (+HS, 35°C for 45 min to 2.5 h), following recovery at 20°C for 2 h. Arrows point to formed granules and quantification of the granule number is shown in the lower panel for each strain. Values on Y axis show the number of granules per head (BRF211), per 100 µM length of excretory cell (BRF255) or per 2500 µm^2^ per worm (mean±SD, see Materials and Methods and Table S4). Asterisks show the intestinal auto-fluorescence. Scale bar: 25 µm.

We next examined the pattern of the GFP fusion transgenes in adult worms subjected to heat or oxidative stress, conditions that elicit SG assembly in mammalian and yeast cells. In contrast to *tiar-3::gfp* protein, which constantly remained nuclear after each stress (data not shown), we observed the formation of numerous cytoplasmic aggregates containing *gfp::tiar-1* in response to heat-shock (HS) and sodium arsenite (SA) (Fig. 6B). In the case of *tiar-2*, the aggregates induced by heat-shock were more visible in the excretory cell of worms, but were also vigorously formed in muscles when we used a strong, muscle-specific promoter (*P_myo-3_::gfp::tiar-2*) to enhance the fluorescent signal and avoid the intestinal autofluorescence at the confocal settings of *gfp::tiar-2* (Fig. 6B). Under oxidative stress we could monitor granules only in muscle-expressed *gfp::tiar-2* (Fig. 6B). The relative mRNA levels of each transgenic line are shown in figure 6C. The heat-shock induced granules, monitored by *gfp::tiar-1* or *gfp::tiar-2*, were not the result of increased transcription of *tiar-1* or *tiar-2*, respectively (Fig. 6D) and were not formed in worms expressing the transcriptional *gfp* fusions (data not shown). In accordance with the dynamic nature of SGs, the above heat-induced granules were formed rapidly (within 45 min) and could be dissociated within 2 h of recovery at 20°C (Fig. 7). Deletion of *tiar-1* did not affect the aggregation of *tiar-2*-containing granules and *vice versa* (data not shown). Moreover, under heat-shock conditions several RFP::TIAR-1 granules contain the IFE-2::GFP and partially colocalise with DCAP-1::GFP granules, while GFP::TIAR-2 also co-localizes partially with DCAP-1::RFP (Fig. S7A–C), again consistent with the spatial overlapping of PBs and SGs [12], [15], [60]. As opposed to PBs, RNAi-mediated depletion of *cgh-1* did not affect the formation of SGs upon heat-shock and *xrn-1(RNAi)* did not lead to accumulation of SGs under normal conditions of growth (data not shown), similar to our observations with IFE-2::GFP marker. Finally, our data using IFE-2 reporter suggest that aging cannot induce the formation of SGs, in sharp contrast to PBs. Indeed, we did not observe an age-dependent accumulation of TIAR proteins in SGs, comparing 6-day to 1-day adult worms at 25°C (Fig. 6B), which show similar expression levels of *tiar-1* or *tiar-2* (data not shown). These data strongly suggest that neither the mild stress accompanying aging nor the age-related alterations in mRNA degradation is sufficient to induce SG formation.

In the process of monitoring the fluorescent *tiar* granules, we noticed a tissue-specific response in their formation under heat-shock, with *gfp::tiar-1* aggregating mostly in head neurons, muscles, intestine, vulval and hypodermal cells, whereas *gfp::tiar-2* granules were visible mainly in excretory cell and less in intestine, muscle and hypodermal cells. Furthermore, by generating transgenic animals co-expressing *rfp::tiar-1* and *gfp::tiar-2* we observed the same tissue-specific aggregates in response to heat-shock, with partial co-localization of the two fusion proteins only in muscles, hypodermal and intestinal cells (Fig. S7D). This implies some tissue-specific functions of each TIAR protein in cellular stress response. In support of this, only TIAR-2-containing granules were formed under osmotic stress (Fig. S8A). In addition, we observed a different requirement on the activity of GCN-2 kinase to initiate the formation of each type of SGs in response to oxidative stress. Having shown the conserved function of GCN-2 as an eIF2α kinase in worms [76] we tested whether SGs were formed in the *gcn-2(ok871)* loss-of-function mutant. While both TIAR-1 and TIAR-2 foci could assemble upon heat-shock in *gcn-2(ok871)*, only TIAR-2 aggregates were formed after treatment with sodium arsenite (Fig. S8B). Thus, TIAR-1 accumulation in response to oxidative stress requires phosphorylation of eIF2α by GCN-2 (and not by the other eIF2α kinase in worms, PEK-1, data not shown). Overall, we showed that the three TIAR proteins in *C. elegans* differ in cellular localization and only TIAR-1 and TIAR-2 participate in cytoplasmic SGs that have both overlapping and distinct patterns, with different assembly requirements depending on the stress stimulus.

### Diverse function of SG-related genes in development, lifespan and stress response

**Figure 8:**
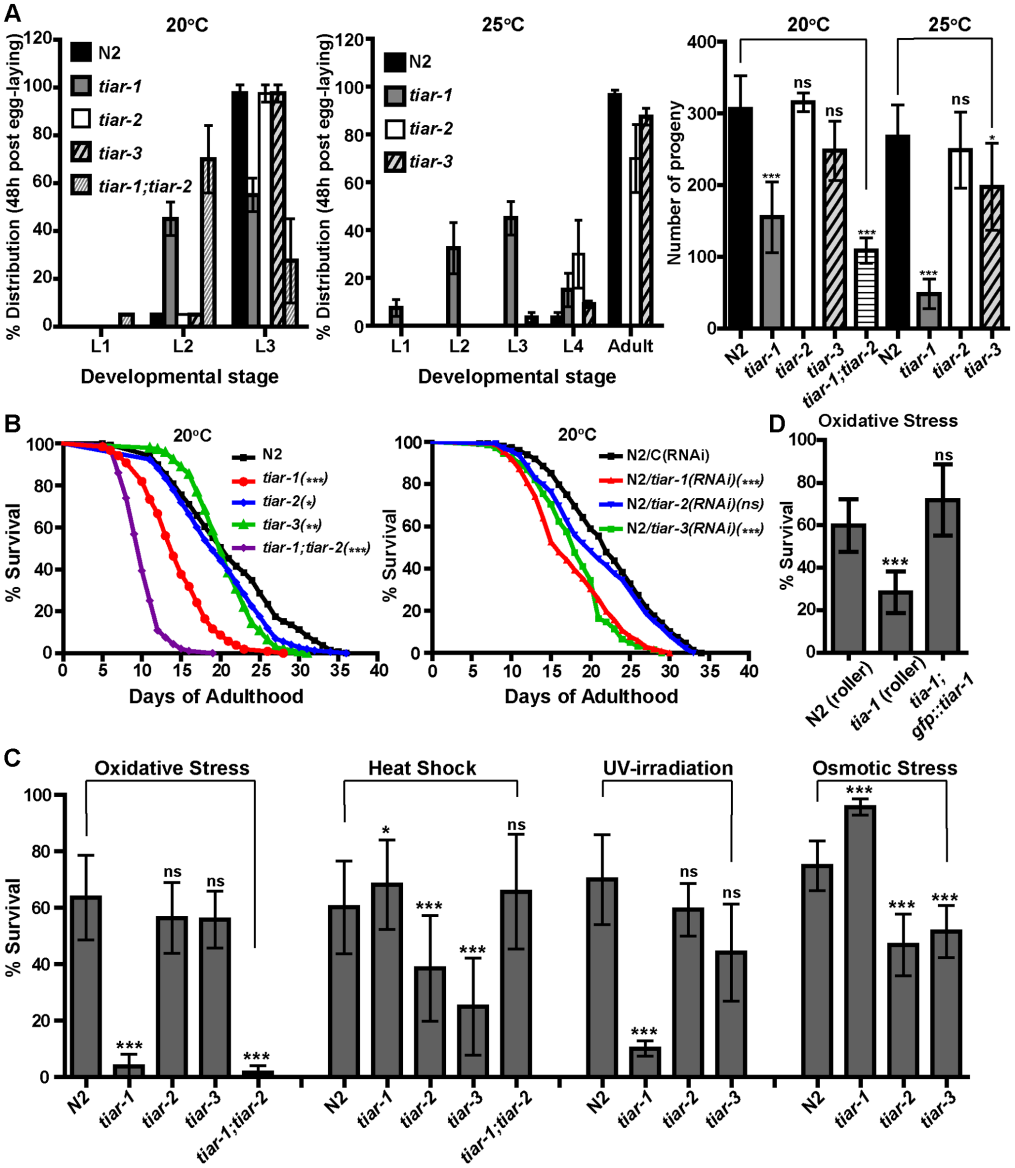
Effects of *tiar* genes deletion on development, fertility, lifespan and stress survival. (A) Developmental rate and brood size of *tiar-1*, *tiar-2*, *tiar-3* and *tiar-1;tiar-2* mutant worms, compared to N2. (B) Survival curves of the above mutants or of N2 worms treated post-developmentally with *tiar-1(RNAi)*, *tiar-2(RNAi)* or *tiar-3(RNAi)*. (C) Survival of adults of the above mutants in oxidative stress (1-day adults at 5 mM sodium arsenite for 48 h), heat-shock (1-day adults at 35°C for 6 h), UV-irradiation (5-day adults at 0.2 J/cm^2^) or osmotic stress (1-day adults at 400 mM NaCl for 24 h, compared to N2. (D) Survival of *tiar-*1 mutants expressing the *gfp::tiar-1* rescuing transgene in oxidative stress (1-day adults at 5 mM sodium arsenite for 24 h) compared to N2 or *tiar-1* animals carrying only the *rol-6(su1006)* roller marker. In lifespan assays the p-values were determined using the log-rank test (see Tables S5 and S6) and in stress assays the error bars show the SD in unpaired *t*-tests (see Materials and Methods). ns indicates not significant (p>0.05); * indicates significant (p-value 0.01 to 0.05); ** indicates very significant (p-value 0.001 to 0.01); *** indicates extremely significant (p<0.001).

Because of the differences in cellular responses of TIAR proteins we tested the impact of each *tiar* gene deletion on development, lifespan and stress survival. We used the mutant strains *tiar-1(tm361), tiar-2(tm2923)* and *tiar-3(ok144)* that harbor internal deletions in the coding region of *tiar* genes and accessed their phenotypes. Mutant *tiar-1* worms exhibited delayed developmental rate and reduced brood size compared to N2, while *tiar-2* and *tiar-3* mutants had milder (at 25°C) or no defect (at 20°C) on both development and fecundity (Fig. 8A). Also, *tiar-1* mutants displayed early behavioral decline, with premature onset of uncoordinated locomotion at day 6 of adulthood (Movies S1 and S2), which was not observed in *tiar-2* and *tiar-3* mutants. Animals lacking both *tiar-1* and *tiar-2* genes (*tiar-1; tiar-2*, Fig. 8A) displayed even slower development than the single mutants, with high matricide deaths at 25°C, suggesting additive effects.

We also assessed differences in the lifespan of *tiar* mutants; deletion of each *tiar* gene shortened normal lifespan, but the effect was more dramatic for *tiar-1* allele (Table S5). Since both the developmental (data not shown) and lifespan (Table S5) defects of *tiar-1* were rescued by the functional GFP::TIAR-1 fusion, these defects are linked to *tm361* allele and not to a secondary mutation. For *tiar-2* allele, there was a temperature-dependent effect on lifespan, as in development, with significantly shorter lifespan at 25°C (Table S5). The double mutant *tiar-1; tiar-2* had an even shorter lifespan than each single mutant at any temperature (Fig. 8B and Table S5). We obtained similar results on N2 lifespan by RNAi of *tiar* genes post-developmentally (Fig. 8B and Table S6). This suggests that the lifespan reduction was not solely due to the developmental abnormalities of each mutant. In addition, by introducing each mutation to the germline-deficient *glp-1(e2141)* worms we observed that loss of *tiar-1* and, to a greater extent, *tiar-2* from somatic cells significantly reduced the long lifespan of *glp-1* worms (Table S5). Surprisingly, deletion of *tiar-3* significantly improved the mean lifespan of *glp-1* worms, contrary to N2 (Table S5). Thus, the impact of *tiar* deletions on lifespan can be influenced by temperature or germline signaling.

Finally, we measured diverse responses of *tiar* mutants against various insults, which are, in some cases, related to their expression pattern. *tiar-1* deletion reduced the survival of worms in sodium arsenite-induced oxidative stress, or after UV-irradiation but increased survival under heat or osmotic stress (Fig. 8C). The impaired oxidative stress response of *tiar-1(tm361)* was rescued by the *gfp::tiar-1* transgene (Fig. 8D). In the imaging analysis we observed TIAR-1-positive granules upon heat and oxidative stress but not under osmotic stress (Fig. 6B and Fig. S8A). We assume that deletion of TIAR-1 affects only specific functions of SGs related to oxidative stress response and not to heat-shock, although it localizes to SGs formed under both conditions. Alternatively, since TIAR-1 is required to induce germ cell apoptosis under various stresses [75], the increased resistance of *tiar-1* mutants to heat-shock might be related to the systemic stress response that is observed in several DNA-damage checkpoint mutants affecting programmed cell death in germline [77], [78]. In sharp contrast to *tiar-1* worms, *tiar-2* or *tiar-3* mutants had increased mortality under heat and osmotic stress but survived as N2 under oxidative or UV-irradiation stress (Fig. 8C). Interestingly, the expression of *tiar-2* driven by its own promoter was strong in the excretory system of worms which has a role in osmoregulation, analogous to the ‘kidney’ of higher animals [79]. TIAR-2 granules formed in oxidative stress, (BRF255 in Fig. 6B), were hardly visible while TIAR-1 granules under the same conditions, were readily formed in all tissues (Fig. 6B), suggesting a more crucial role of TIAR-1 under this stress. The double mutant *tiar-1; tiar-2* survived similar to the single *tiar-1*, at least under heat and oxidative stress (Fig. 8C). While further studies are required to address these issues, our data show that the three *tiar* genes exhibit diverse developmental, lifespan and stress response outputs at the organismal level that are possibly reflected by their specific functions, which are differentially regulated under various cellular contexts and stressors.

## Discussion

Post-transcriptional regulation of eukaryotic gene expression is attained through several mRNA-specific control mechanisms, such as processing, export, turnover and translation. PBs and SGs are cytoplasmic aggregates of RNP complexes that mediate the subcellular localization, translation or decay of bulk mRNA in eukaryotic cells [6], [8], [11]. PBs consist of mRNAs associated with translation repressors or the 5' to 3' mRNA decay machinery, whereas SGs contain a pool of mRNAs stalled in the process of translation initiation in response to stress. Both are dynamic structures that transiently interact and share many components [12]. Hence, PBs and SGs are considered as hubs of mRNP trafficking that determine the fate of mRNAs but also as regulators of stress-responsive signaling pathways that determine the survival of cells under stress [80]. Although the function of these granules provides another level for fine-tuning gene expression to maintain cellular and tissue homeostasis, studies addressing their impact or pattern during stress or aging, at the level of intact organism, are limited. In *C. elegans*, related RNA-protein particles function in maternal mRNA metabolism and have been well-studied in germline development and embryogenesis [30]–[32], [34], [36].

Here, we documented the formation of PBs and SGs in somatic cells of *C. elegans* by using fluorescent reporters of known components of these granules. These include the decapping complex subunit DCAP-1/DCP1 and the miRNA-binding protein AIN-1/GW182, as PB markers and the homologues of human TIA-1/TIAR RNA-binding proteins, TIAR-1-to-3, as SG markers. Of the three worm TIAR proteins, only TIAR-1 and TIAR-2 contain a QN-rich C′-terminal domain and are distributed in both nucleus and cytoplasm. As shown by live imaging in adult transgenic worms there were no SGs and few PBs under normal conditions but there was robust aggregation of PB and SG reporters in the cytoplasm of most cells/tissues, in response to heat or oxidative stress. The dynamic nature of these aggregates is reminiscent of yeast and mammalian PBs and SGs; their formation is rapid and reversible, whereas the partial co-localization between the heat-induced DCAP-1 and TIAR-1 or TIAR-2 foci indicates that PBs and SGs could overlap or dock to each other in worms [12], [15], [49], [51], [60]. Additionally, we showed that the translation initiation factor IFE-2/eIF4E is not a component of constitutive PBs and localizes mainly to SGs in response to stress. The heat-induced accumulation of PBs, as monitored by DCAP-1::GFP, was affected by alterations in genes influencing PB assembly in other organisms; it was induced by RNAi of the 5′ to 3′ exonuclease *xrn-1* and prevented by depletion of the translation regulator *cgh-1*/RCK (Dhh1 in yeast). In *C. elegans* gonad, loss of *cgh-1*induces the formation of aberrant sheet-like structures through the relocalization of various PB components [34], [35], [81], [82]. Interestingly, we did not observe such structures in somatic tissues of *cgh-1(RNAi)*-treated animals; instead PBs aggregation was prevented even upon stress. Possible reasons for this discrepancy could be related to different tissues or markers that we used since it has been suggested that the function of CGH-1 helicase depends on cellular and developmental context [34] and *cgh-1* loss induces relocalization of a subset of RNP factors (as CAR-1) into sheet-like structures but dissociates PATR-1 or PGL-1 [82], [83]. Furthermore, the fact that knockdown of *cgh-1* prevents PB accumulation without affecting SG formation under stress, suggests that SGs can form independently of PBs. This supports evidence in yeast and mammalian cells, indicating that assembly of PBs and SGs is regulated by different signaling pathways [12], [27], [60], [84].

We also investigated the impact of aging on the pattern of somatic PBs and SGs in *C. elegans*. We demonstrated that only PBs accumulated with age, showing an increase in their number and size. In sharp contrast, we did not observe SG formation in aged animals, despite that in aged oocytes both PB and SG components co-localize to large RNP structures [33]. Since IFE-2 and TIAR-1/-2 were localized in cytoplasmic aggregates induced by sodium arsenite, we reasoned that the accumulation of PBs with age does not result from oxidative stress in aged tissues. The accumulation of non-translatable mRNAs into PBs, due to reduction of either translation or degradation rates with age, provides another possible explanation for the increased PB aggregation during aging. However, knockdown of several translation factors or deletion of *ife-2* did not induce the formation of DCAP-1::GFP granules in adult worms, in contrast to the increased number and size of PBs caused by *xrn-1(RNAi)*. Similarly to the age-induced aggregates, the *xrn-1(RNAi)*-induced PBs did not contain SG markers (IFE-2 or TIAR-1/-2). Thus, we presume that the increased formation of PBs in aged tissues could be an outcome of age-related alterations in decay rates rather than translation but further experimentation is required to support this hypothesis. Alternatively, the accumulation of PBs in older ages could be just a consequence of the aging process, which is associated with a large increase in protein insolubility and aggregation of diverse proteins [85], [86]. Among these are translation factors, ribosomal subunits and proteins involved in mitochondrial respiration, whose inhibition has been linked to longevity in many organisms [1]. Thus, we examined the effects of direct alterations in PB components on lifespan. In contrast to the above factors, post-developmental RNAi of genes related to PBs significantly shortened lifespan of N2 and several long-lived mutants. Moreover, increased expression of *dcap-1* in somatic cells of young adults improved their resistance in both heat and oxidative stress.

The maintenance of protein homeostasis, through regulation of both translation and protein degradation is considered a common longevity assurance mechanism [87]. Our findings support that mRNA metabolism factors contribute to such mechanisms and impaired function of PBs can limit lifespan. Interestingly, studies in *S. cerevisiae* have demonstrated that PBs are required for the long-term survival of stationary phase cells [88]. PBs functions are also vital for normal development and stress management. Mutant worms for the decapping genes exhibited severe defects in development, growth, fecundity, movement and impaired stress responses to a variety of environmental insults. A role in the protection of the nascent germline from stress has been described for P granules in worms [89]. Noteworthy, we noticed that *dcap-1* is highly expressed in the three pairs of coelomocytes, the scavenger cells that are considered as a primitive immune system in *C. elegans*. Moreover, we revealed diverse roles of worm TIAR proteins in the cellular stress response, as they displayed specific expression pattern and assembly requirements that can differ according to the stress stimulus. Consistent with such diverse roles, we measured differences in development, growth and lifespan of worms carrying mutations in any of the three *tiar* genes. The effects of *tiar* alleles on lifespan were influenced by temperature or germline signaling; loss of each *tiar* gene shortened normal lifespan, mainly at 25°C, whereas in the long-lived, germline-deficient mutant *glp-1* loss of *tiar-3* resulted in increased mean lifespan, in contrast to *tiar-1* or *tiar-2* deletion that reduced its longevity. The latter indicates a currently unknown role of the predicted RNA-binding protein TIAR-3 in germline-mediated longevity. We finally demonstrated diverse responses of *tiar* mutants against various stressors, suggesting different activities for these genes in worms. Such diverse cellular roles have been described for several RNA-binding proteins which regulate various mRNA subpopulations, often in a coordinated manner [90], [91]. For example, several hundred mRNAs of various functions were identified as targets of the dsRNA binding protein STAU-1, the worm homologue of human and *Drosophila* Staufen proteins that are localized in neuronal RNPs and SGs under stress [92]–[94]. In conclusion, our work implicates factors related to PBs and SGs in the normal lifespan and stress responses, beyond their developmental roles in *C. elegans*. Our observations expand our knowledge on the formation, function and relationship between PBs and SGs in the somatic cells of worms and reveal their tissue- and stress-specific properties.

## Supporting Information

Figure S1 **Expression pattern and mRNA levels of** ***dcap-1*** **in the used transgenic lines.** (A) Representative confocal images of 1-day adult worms expressing the transcriptional fusion *P_dcap-1::gfp_* (BRF154) or the translational fusion *dcap-1::gfp* in N2 (BRF155 and BRF261) or germline-deficient *glp-1(e2141)* worms (BRF219), normally grown at 25°C. m: muscles, n: neurons, sp: spermatheca, exc: excretory cell, v: vulva, i: intestine, cc: coeloemocytes. Scale bar: 50 µm. (B) Expression levels of *dcap-1* gene in N2, *glp-1(e2141)* or the indicated transgenic strains measured by quantitative RT-PCR and normalized to *ama-1(mRNA)* levels. Error bars show the SD of the means of two independent experiments.
(TIF)

Figure S2 **Monitoring of granule formation under oxidative stress, heat-shock or aging by using other reporter markers.** (A) Representative confocal images of 1-day adults expressing the translational fusion *dcap-1::gfp* (BRF155 in Table S1) or *ife-2::gfp* (BRF70 in Table S1) under normal conditions (-SA) or after exposure to 10 mM sodium arsenite for 3 h (+SA) at 25°C. (B) Representative confocal images of 1-day adults expressing the translational fusion *ain-1::gfp* (MH2704 in Table S1), the transcriptional fusion of *ife-2* putative promoter to *gfp* (BRF68 in Table S1) or the translational fusion *ife-2::gfp* (BRF70 in Table S1) under normal conditions (-HS) or after heat-shock at 35°C for 3 h (+HS). The same transgenic animals are shown at the day 5 of adulthood, grown under normal conditions, at 25°C. (C) Representative confocal images of 1-day adults co-expressing *ife-2::gfp* and *dcap-1::rfp* (BRF313 in Table S1). Arrows point to induced granules. Scale bar: 25 µm. (D) Expression levels of *ain-1* gene in N2, as 1-day or 5-day adults grown under normal conditions (-HS) or as 1-day adults exposed to heat-shock at 35°C for 3 h (+HS), measured by quantitative RT-PCR and normalized to *ama-1(mRNA)* levels. Error bars show the SD of the means of two independent experiments.
(TIF)

Figure S3 **Effects of** ***cgh-1(RNAi)*** **and** ***ife-2*** **deletion in heat-induced granule formation.** (A) Representative confocal images of 1-day adults expressing the translational fusion *ife-2::gfp* (BRF70 in Table S1) fed from eggs either control Control(RNAi) or *cgh-1(RNAi)* bacteria, at 25°C, under normal conditions (-HS) or after heat-shock at 35°C for 3 h (+HS). (B) Representative confocal images of 1-day adults expressing the translational fusion *dcap-1::gfp* in N2 (BRF155 in Table S1) or in *ife-2(ok306)* mutant background (BRF220 in Table S1), grown under normal conditions (-HS) or after heat-shock at 35°C for 3 h (+HS). Arrows point to induced granules. Scale bar: 25 µm.
(TIF)

Figure S4 **Accumulation of PBs with age at 20°C.** (A) Representative confocal images of 1-day, 5-day, 10-day and 15-day adults grown at 20°C, expressing the translational fusion *dcap-1::gfp* (BRF155). Arrows point to DCAP-1::GFP granules. Scale bar: 25 µm. (B) Quantification of data presented in (A). Values on Y axis show the number of granules per head.
(TIF)

Figure S5 **Decapping genes are important for development, growth, fertility and stress response.** (A) Distribution of N2, *dcap-1* and *dcap-2* progeny in the indicated developmental stages (L1-L4 and adult) 48 h after egg-laying, at 20°C and 25°C. (B) Number of *dcap-1* and *dcap-2* progeny (brood size), compared to N2, at 20°C and 25°C. (C) Development and stress resistance of *dcap-1* mutants expressing the *dcap-1::gfp* rescuing transgene, compared to N2 and *dcap-1* worms carrying only the rol-6(su1006) roller marker, at 20°C. For stress assays see Materials and Methods. Error bars show the SD in unpaired t-tests. ns indicates not significant (p>0.05); ** indicates very significant (p-value 0.001 to 0.01); *** indicates extremely significant (p<0.001). (D) Reverse transcription (RT)-PCR of *dcap-1(mRNA)* in 1-day adult N2 and *dcap-1* mutants and alignment of the wild-type DCAP-1 protein and the one encoded by the *tm3163* allele using MultAlin (http://multalin.toulouse.inra.fr/multalin/multalin.html).
(TIF)

Figure S6 **Expression pattern of** ***tiar*** **genes in the used transgenic lines.** (A) Representative confocal images of 1-day adult worms expressing the transcriptional fusion *P_tiar-1_::gfp* (BRF118 in Table S1), the translational fusion *gfp::tiar-1* (BRF211 in Table S1), the transcriptional fusion *P_tiar-2_::gfp* (BRF238 in Table S1), the translational fusion *gfp::tiar-2* (BRF255 in Table S1) or the translational fusion *tiar-3::gfp* (BRF120 in Table S1), normally grown at 20°C. m: muscles, n: neurons, sp: spermatheca, exc: excretory cell, v: vulva, i: intestine, cc: coeloemocytes. Scale bar: 50 µm.
(TIF)

Figure S7 **Spatial overlapping of PB and SG components in somatic cells of worms.** Representative confocal images of 1-day adults, under normal conditions (-HS) or after heat-shock at 35°C for 3 h (+HS), co-expressing: (A) *rfp::tiar-1* and *ife-2::gfp* (BRF312 in Table S1), (B) *rfp::tiar-1* and *dcap-1::gfp* (BRF328 in Table S1), (C) *gfp::tiar-2* and *dcap-1::rfp* (BRF369 in Table S1), (D) *rfp::tiar-1* and *gfp::tiar-2* (BRF361 in Table S1). B and C are confocal optical sections. All fusions are driven by their own promoters. Arrows point to induced granules. Scale bar: 25 µm.
(TIF)

Figure S8 **TIAR-1 and TIAR-2 granules show differences in their formation.** Representative confocal images of 1-day adults expressing (A) *gfp::tiar-1* (BRF211 in Table S1), *gfp::tiar-2* (BRF255 in Table S1) or *P_myo-3_::gfp::tiar-2* (BRF310 in Table S1) subjected to 350 mM NaCl and visualized after 0.5–1 h, (B) *gfp::tiar-1* or *P_myo-3_::gfp::tiar-2* in N2 (BRF211 or BRF310, respectively in Table S1) and *gcn-2(ok871)* background (BRF294 or BRF339, respectively in Table S1), subjected to heat-shock (HS, 35°C for 3 h) or sodium arsenite (SA, 15 mM for 3 h). Arrows point to induced granules. Scale bar: 25 µm.
(TIF)

Table S1 **List of the strains used in this study.**
(DOCX)

Table S2 **Primers used in this study.**
(DOCX)

Table S3 **RNAi plasmids used in this study.**
(DOCX)

Table S4 **Quantification of number of granules.**
(DOCX)

Table S5 **Lifespan assays in OP-50 plates.**
(DOCX)

Table S6 **Lifespan assays in RNAi plates.**
(DOCX)

Movie S1 **Motility of 6-day adults of N2 on OP-50 plates, at 25°C.** Movie was taken in a Leica M205 FA fluorescence stereoscope with a Leica DFC340 FX camera.
(MPG)

Movie S2 **Motility of 6-day adults of** ***tiar-1(tm361)*** **on OP-50 plates, at 25°C.** Movie was taken in a Leica M205 FA fluorescence stereoscope with a Leica DFC340 FX camera.
(MPG)
