# Supplementary figures and images for: Diverse Functions of mRNA Metabolism Factors in Stress Defense and Aging of Caenorhabditis elegans
Source: PLoS One. 2014 Jul 25;9(7):e103365. doi: 10.1371/journal.pone.0103365 (PMC4111499; doi:10.1371/journal.pone.0103365)

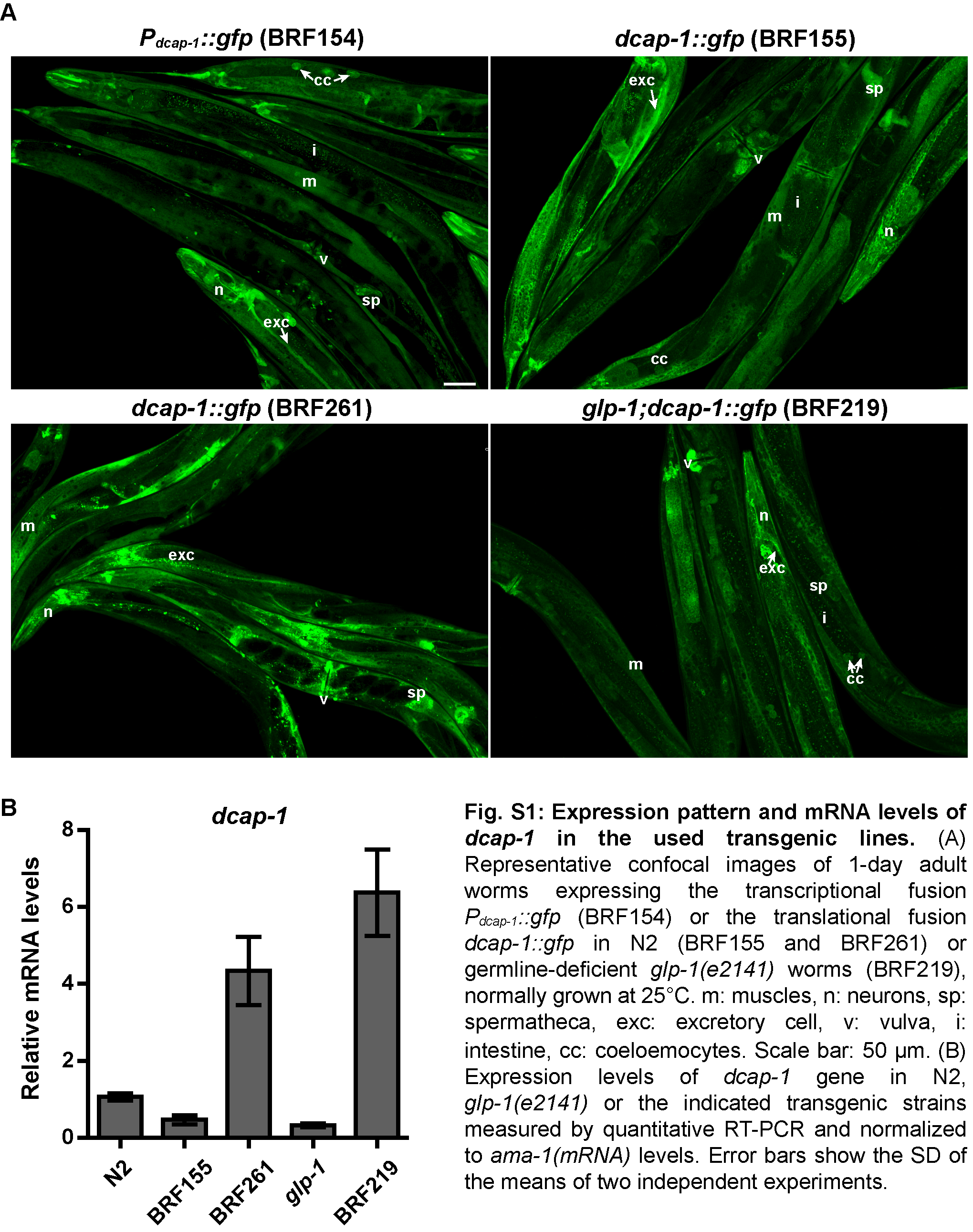

Supplement: Figure S1 — Expression pattern and mRNA levels of dcap-1 in the used transgenic lines. (A) Representative confocal images of 1-day adult worms expressing the transcriptional fusion Pdcap-1::gfp (BRF154) or the translational fusion dcap-1::gfp in N2 (BRF155 and BRF261) or germline-deficient glp-1(e2141) worms (BRF219), normally grown at 25°C. m: muscles, n: neurons, sp: spermatheca, exc: excretory cell, v: vulva, i: intestine, cc: coeloemocytes. Scale bar: 50 µm. (B) Expression levels of dcap-1 gene in N2, glp-1(e2141) or the indicated transgenic strains measured by quantitative RT-PCR and normalized to ama-1(mRNA) levels. Error bars show the SD of the means of two independent experiments. (TIF) [file pone.0103365.s001.tif]

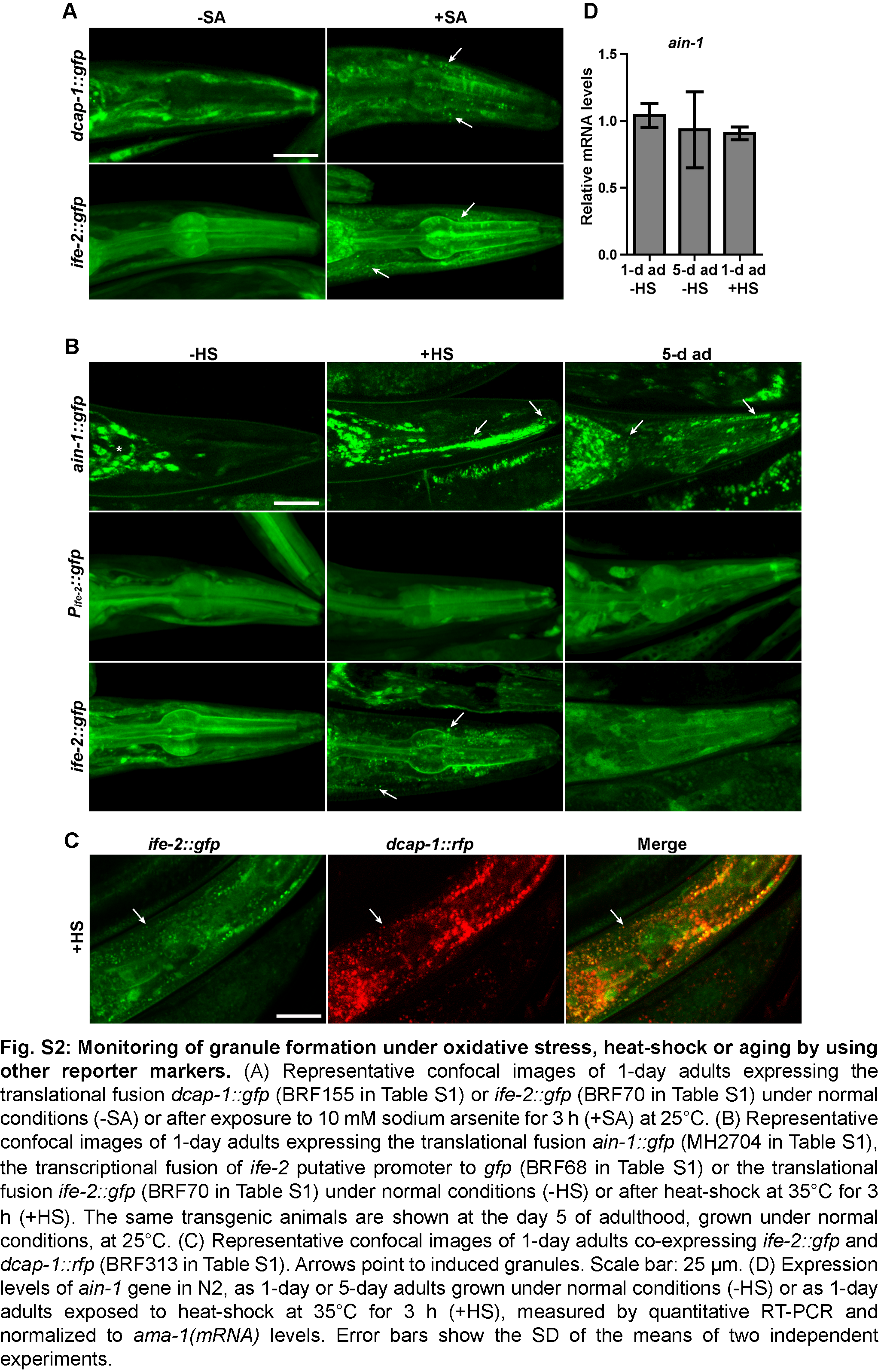

Supplement: Figure S2 — Monitoring of granule formation under oxidative stress, heat-shock or aging by using other reporter markers. (A) Representative confocal images of 1-day adults expressing the translational fusion dcap-1::gfp (BRF155 in Table S1) or ife-2::gfp (BRF70 in Table S1) under normal conditions (-SA) or after exposure to 10 mM sodium arsenite for 3 h (+SA) at 25°C. (B) Representative confocal images of 1-day adults expressing the translational fusion ain-1::gfp (MH2704 in Table S1), the transcriptional fusion of ife-2 putative promoter to gfp (BRF68 in Table S1) or the translational fusion ife-2::gfp (BRF70 in Table S1) under normal conditions (-HS) or after heat-shock at 35°C for 3 h (+HS). The same transgenic animals are shown at the day 5 of adulthood, grown under normal conditions, at 25°C. (C) Representative confocal images of 1-day adults co-expressing ife-2::gfp and dcap-1::rfp (BRF313 in Table S1). Arrows point to induced granules. Scale bar: 25 µm. (D) Expression levels of ain-1 gene in N2, as 1-day or 5-day adults grown under normal conditions (-HS) or as 1-day adults exposed to heat-shock at 35°C for 3 h (+HS), measured by quantitative RT-PCR and normalized to ama-1(mRNA) levels. Error bars show the SD of the means of two independent experiments. (TIF) [file pone.0103365.s002.tif]

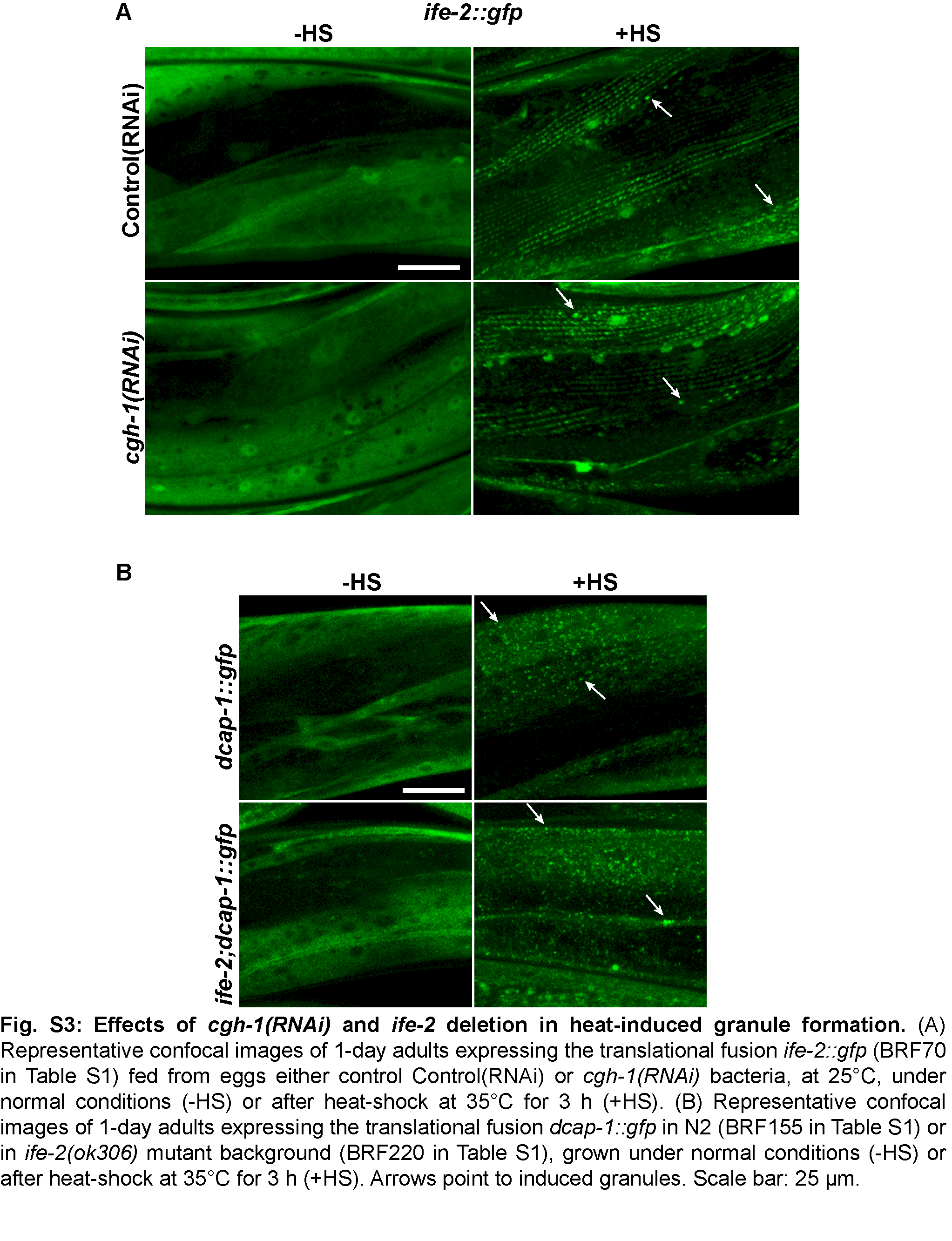

Supplement: Figure S3 — Effects of cgh-1(RNAi) and ife-2 deletion in heat-induced granule formation. (A) Representative confocal images of 1-day adults expressing the translational fusion ife-2::gfp (BRF70 in Table S1) fed from eggs either control Control(RNAi) or cgh-1(RNAi) bacteria, at 25°C, under normal conditions (-HS) or after heat-shock at 35°C for 3 h (+HS). (B) Representative confocal images of 1-day adults expressing the translational fusion dcap-1::gfp in N2 (BRF155 in Table S1) or in ife-2(ok306) mutant background (BRF220 in Table S1), grown under normal conditions (-HS) or after heat-shock at 35°C for 3 h (+HS). Arrows point to induced granules. Scale bar: 25 µm. (TIF) [file pone.0103365.s003.tif]

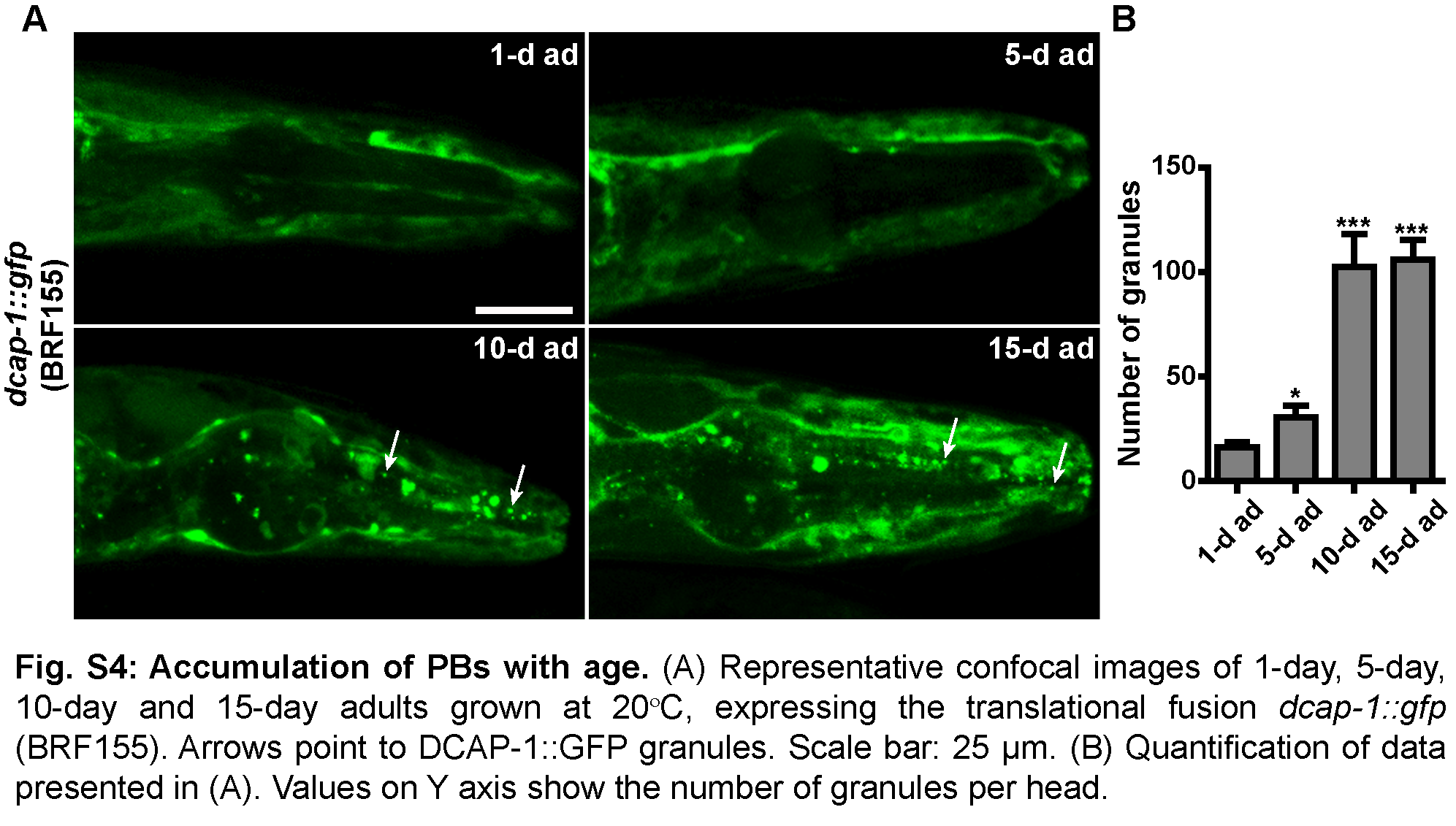

Supplement: Figure S4 — Accumulation of PBs with age at 20°C. (A) Representative confocal images of 1-day, 5-day, 10-day and 15-day adults grown at 20°C, expressing the translational fusion dcap-1::gfp (BRF155). Arrows point to DCAP-1::GFP granules. Scale bar: 25 µm. (B) Quantification of data presented in (A). Values on Y axis show the number of granules per head. (TIF) [file pone.0103365.s004.tif]

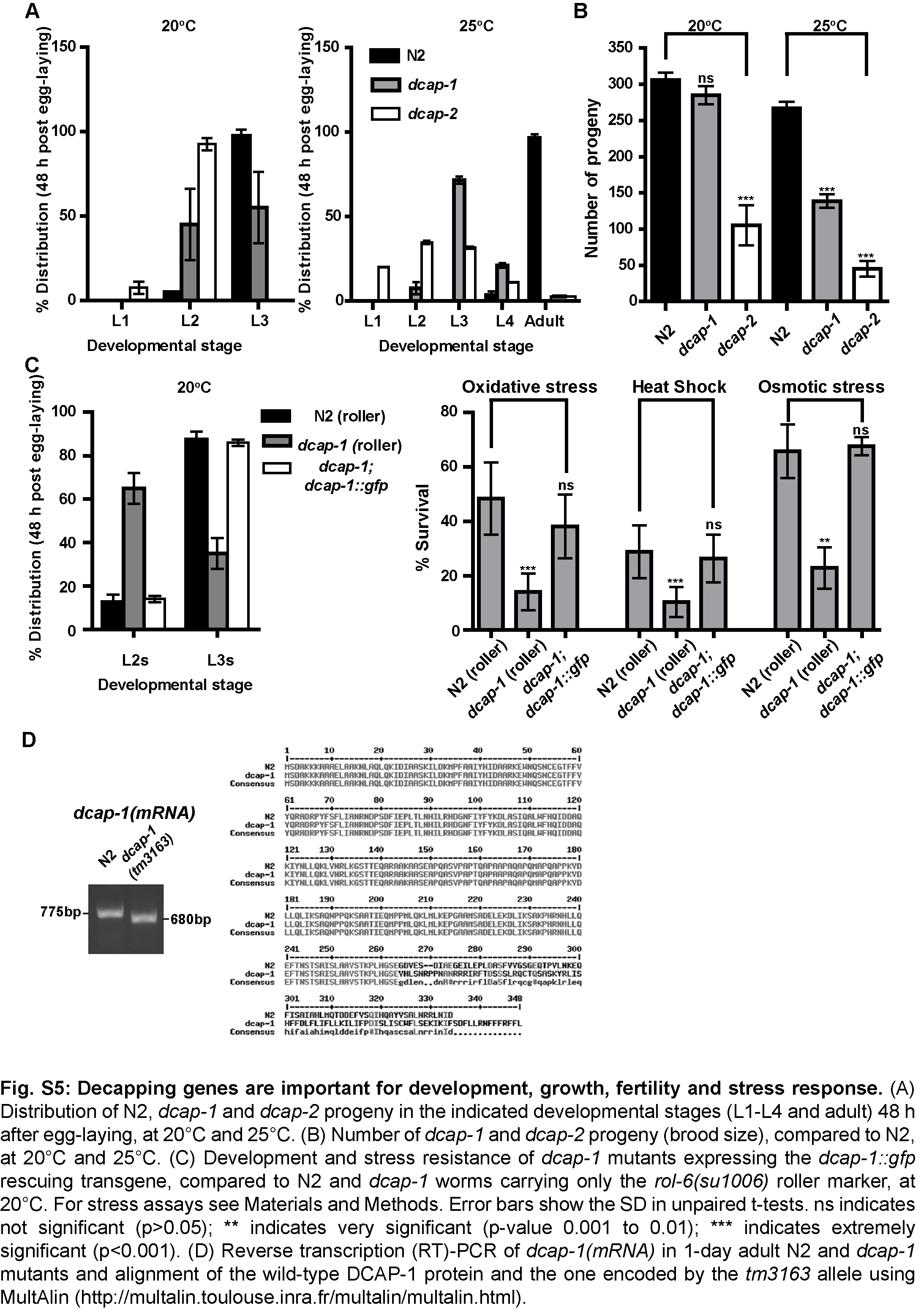

Supplement: Figure S5 — Decapping genes are important for development, growth, fertility and stress response. (A) Distribution of N2, dcap-1 and dcap-2 progeny in the indicated developmental stages (L1-L4 and adult) 48 h after egg-laying, at 20°C and 25°C. (B) Number of dcap-1 and dcap-2 progeny (brood size), compared to N2, at 20°C and 25°C. (C) Development and stress resistance of dcap-1 mutants expressing the dcap-1::gfp rescuing transgene, compared to N2 and dcap-1 worms carrying only the rol-6(su1006) roller marker, at 20°C. For stress assays see Materials and Methods. Error bars show the SD in unpaired t-tests. ns indicates not significant (p>0.05); ** indicates very significant (p-value 0.001 to 0.01); *** indicates extremely significant (p<0.001). (D) Reverse transcription (RT)-PCR of dcap-1(mRNA) in 1-day adult N2 and dcap-1 mutants and alignment of the wild-type DCAP-1 protein and the one encoded by the tm3163 allele using MultAlin (http://multalin.toulouse.inra.fr/multalin/multalin.html). (TIF) [file pone.0103365.s005.tif]

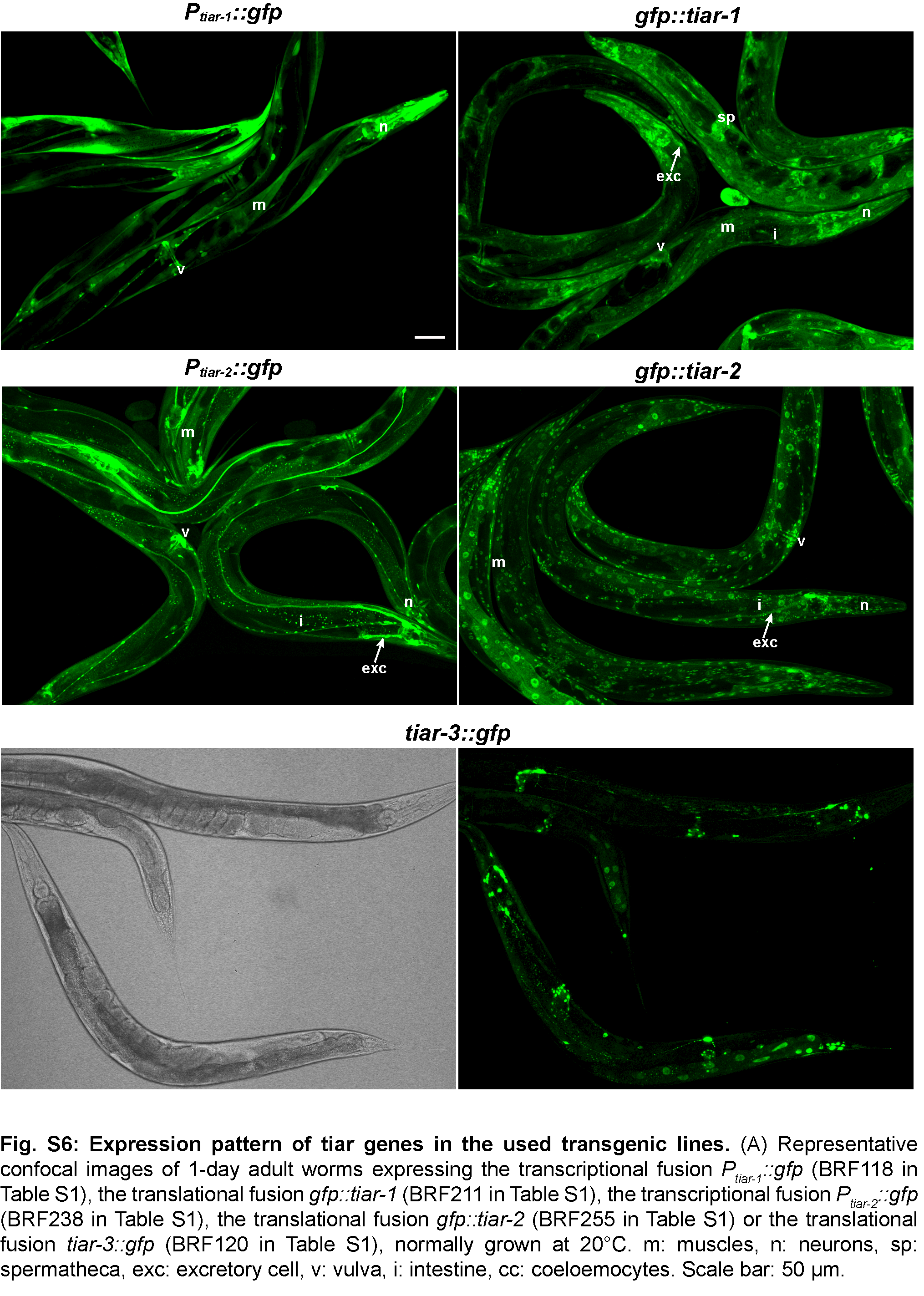

Supplement: Figure S6 — Expression pattern of tiar genes in the used transgenic lines. (A) Representative confocal images of 1-day adult worms expressing the transcriptional fusion Ptiar-1::gfp (BRF118 in Table S1), the translational fusion gfp::tiar-1 (BRF211 in Table S1), the transcriptional fusion Ptiar-2::gfp (BRF238 in Table S1), the translational fusion gfp::tiar-2 (BRF255 in Table S1) or the translational fusion tiar-3::gfp (BRF120 in Table S1), normally grown at 20°C. m: muscles, n: neurons, sp: spermatheca, exc: excretory cell, v: vulva, i: intestine, cc: coeloemocytes. Scale bar: 50 µm. (TIF) [file pone.0103365.s006.tif]

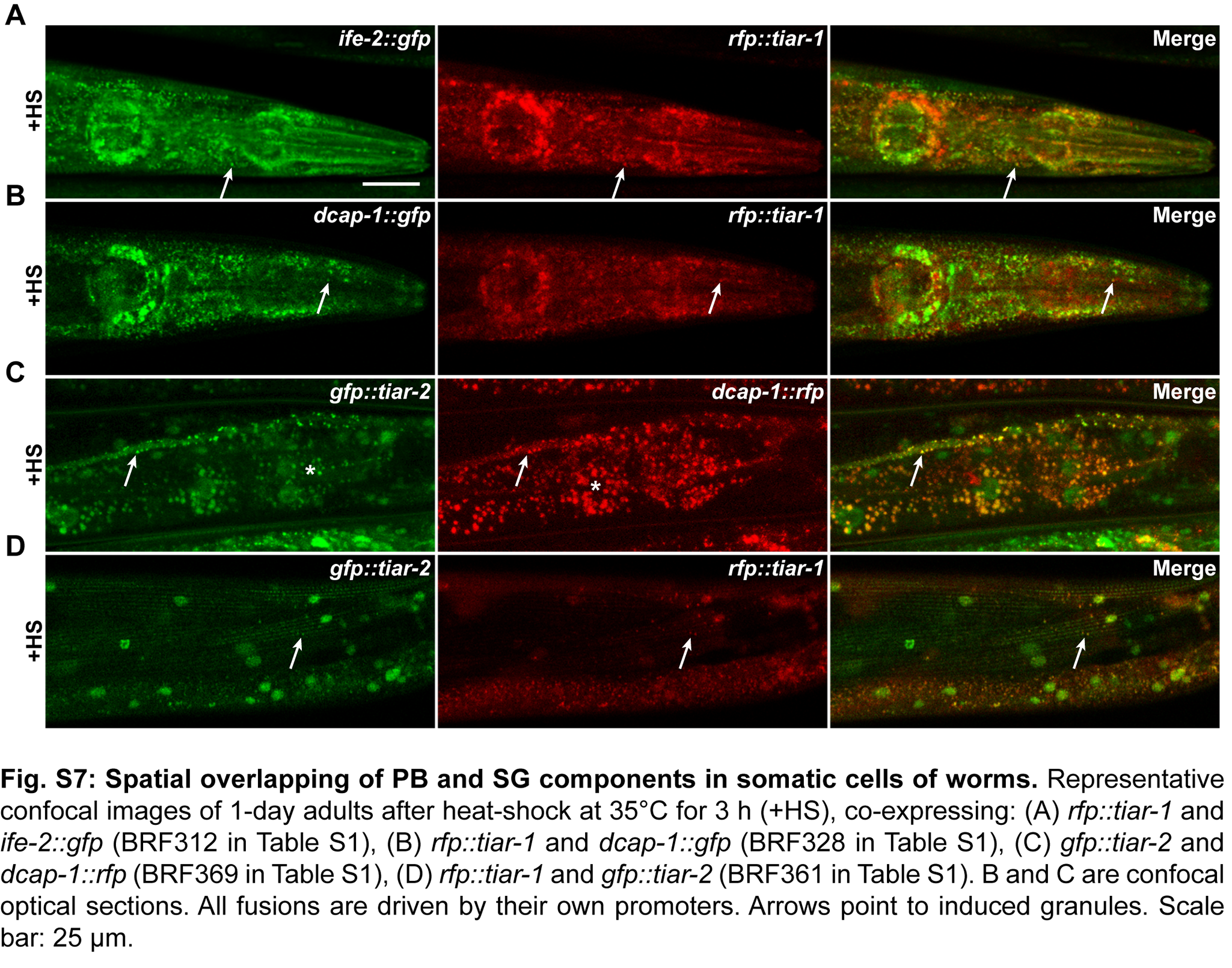

Supplement: Figure S7 — Spatial overlapping of PB and SG components in somatic cells of worms. Representative confocal images of 1-day adults, under normal conditions (-HS) or after heat-shock at 35°C for 3 h (+HS), co-expressing: (A) rfp::tiar-1 and ife-2::gfp (BRF312 in Table S1), (B) rfp::tiar-1 and dcap-1::gfp (BRF328 in Table S1), (C) gfp::tiar-2 and dcap-1::rfp (BRF369 in Table S1), (D) rfp::tiar-1 and gfp::tiar-2 (BRF361 in Table S1). B and C are confocal optical sections. All fusions are driven by their own promoters. Arrows point to induced granules. Scale bar: 25 µm. (TIF) [file pone.0103365.s007.tif]

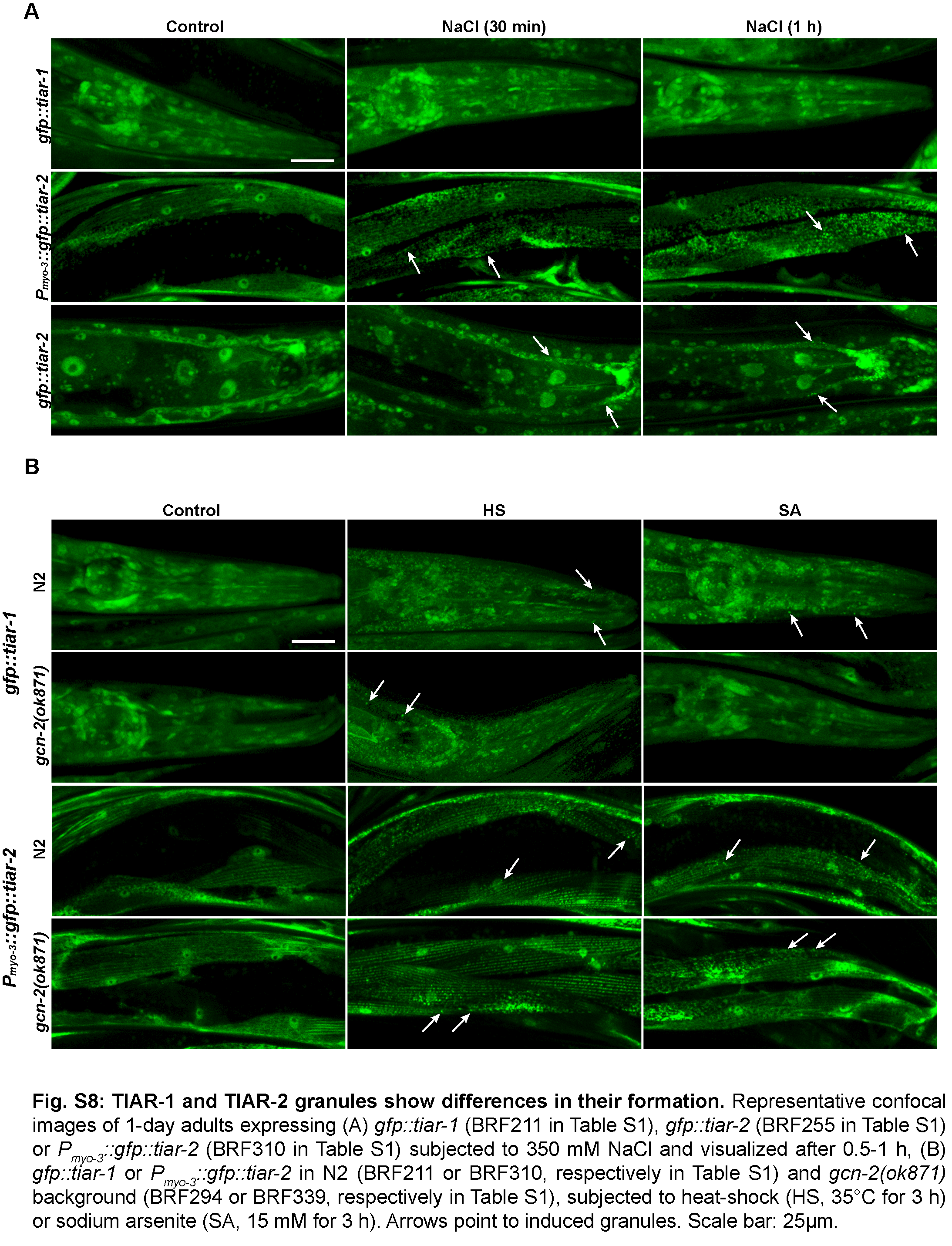

Supplement: Figure S8 — TIAR-1 and TIAR-2 granules show differences in their formation. Representative confocal images of 1-day adults expressing (A) gfp::tiar-1 (BRF211 in Table S1), gfp::tiar-2 (BRF255 in Table S1) or Pmyo-3::gfp::tiar-2 (BRF310 in Table S1) subjected to 350 mM NaCl and visualized after 0.5–1 h, (B) gfp::tiar-1 or Pmyo-3::gfp::tiar-2 in N2 (BRF211 or BRF310, respectively in Table S1) and gcn-2(ok871) background (BRF294 or BRF339, respectively in Table S1), subjected to heat-shock (HS, 35°C for 3 h) or sodium arsenite (SA, 15 mM for 3 h). Arrows point to induced granules. Scale bar: 25 µm. (TIF) [file pone.0103365.s008.tif]
