# Supplementary material for: Diverse Functions of mRNA Metabolism Factors in Stress Defense and Aging of Caenorhabditis elegans
Source: PLoS One. 2014 Jul 25;9(7):e103365. doi: 10.1371/journal.pone.0103365 (PMC4111499; doi:10.1371/journal.pone.0103365)
Supplement: Table S1 — List of the strains used in this study. (DOCX) [file pone.0103365.s009.docx]

**Table S1:** List of the strains used in this study

| **Strain** | **Genotype** | **Information** | **Reference** |
| --- | --- | --- | --- |
| N2 | N2 (Bristol) | Wild-type | CGC |
| BRF159 | *dcap-1(tm3163)IV* | FX03163 (NBRP) backcrossed to N2 5x | This study |
| BRF234 | *dcap-2(ok2023)IV* | RB1641 (CGC) backcrossed to N2 3x | This study |
| BRF161 | *tiar-1(tm361)II* | FX00361 (NBRP) backcrossed to N2 5x | This study |
| BRF171 | *tiar-2(tm2923)II* | FX02923 (NBRP) backcrossed to N2 4x | This study |
| BRF235 | *tiar-3(ok144)X* | BS3348 (CGC) backcrossed to N2 3x | This study |
| BRF214 | *tiar-1(tm361)II tiar-2(tm2923)II* | From crossing of BRF171 males with BRF161 | This study |
| BRF340 | *glp-1(e2141)III* | CF1903(CGC) backcrossed to N2 1x, germline-deficient (ts), long-lived | This study |
| BRF217 | *glp-1(e2141)III; dcap-1(tm3163)IV* | From crossing of BRF340 males with BRF159 | This study |
| BRF252 | *glp-1(e2141)III; dcap-2(ok2023)IV* | From crossing of BRF340 males with BRF234 | This study |
| BRF358 | *glp-1(e2141)III; tiar-1(tm361)II* | From crossing of BRF161 males with BRF340 | This study |
| BRF256 | *glp-1(e2141)III; tiar-2(tm2923)II* | From crossing of BRF340 males with BRF171 | This study |
| BRF240 | *glp-1(e2141)III; tiar-3(ok144)X* | From crossing of BRF340 males with BRF235 | This study |
| CB1370 | *daf-2(1370)III* | Long-lived mutant of the insulin receptor *daf-2* (Class II allele) | CGC |
| DR1572 | *daf-2(1368)III* | Long-lived mutant of the insulin receptor *daf-2* (Class I allele) | CGC |
| BRF302 | *daf-2(e1368)III; dcap-1(tm3163)IV* | From crossing of BRF159 males with DR1572 hermaphrodites | This study |
| BRF306 | *daf-2(e1368)III; dcap-2(ok2023)IV* | From crossing of DR1572 males with BRF234 hermaphrodites | This study |
| DA465 | *eat-2(ad465)II* | Abnormal feeding, long-lived | CGC |
| BRF307 | *eat-2(ad465)II; dcap-1(tm3163)IV* | From crossing of BRF159 males with DA465 hermaphrodites | This study |
| BRF304 | *eat-2(ad465)II; dcap-2(ok2023)IV* | From crossing of DA465 males with BRF234 hermaphrodites | This study |
| KX15 | *ife-2(ok306)X* | Deletion of the major somatic isoform eIF4E, long-lived | CGC |
| BRF179 | *ife-2(ok306)X; dcap-1(tm3163)IV* | From crossing of KX15 males with BRF159 hermaphrodites | This study |
| BRF314 | *ife-2(ok306)X; dcap-2(ok2023)IV* | From crossing of KX15 males with BRF234 hermaphrodites | This study |
| RB1206 | *rsks-1(ok1255)III* | Deletion of the S6K, long-lived | CGC |
| BRF154 | N2; *synEx164[P_dcap-1_::gfp::3'UTR^dcap-1^ ;rol-6(su1006)]* | Transcriptional fusion of *dcap-1* with *gfp* in N2 background | This study |
| BRF155 | N2; *synEx190[P_dcap-1_::dcap-1::gfp::3'UTR^dcap-1^ ;rol-6(su1006)]* | Translational fusion of *dcap-1* with *gfp* in N2 background | This study |
| BRF261 | N2; *synEx191[P_dcap-1_::dcap-1::gfp::3'UTR^dcap-1^ ; rol-6(su1006)]* | Translational fusion of *dcap-1* with *gfp* (vector-free) in N2 | This study |
| BRF219 | *glp-1(e2141); synEx190* | From crossing BRF340 males with BRF155 hermaphrodites | This study |
| MH2704 | *unc-119(ed3); In[P_ain-1_::ain-1::gfp; P_dcap-1_::flag::dcap-1; unc-119(+)]* | Translational fusions of *ain-1* with *gfp* and *flag::dcap-1* | Ding et al., 2005 |
| BRF68 | N2; *synEx114[P_ife-2_::gfp::3'UTR^unc-54^ ;rol-6(su1006)]* | Transcriptional fusion of *ife-2* with *gfp* in N2 background | Syntichaki et al., 2007 |
| BRF70 | N2*; synEx115[P_ife-2_::ife-2::gfp::3'UTR^unc-54^ ;rol-6(su1006)]* | Translational fusion of *ife-2* with *gfp* in N2 background | Syntichaki et al., 2007 |
| BRF313 | N2; *synEx313[P_dcap-1_::dcap-1::tagrfp::3'UTR^unc-54^ ; P_ife-2::_ife-2::gfp::3'UTR^unc-54^ ;rol-6(su1006)]* | Translational fusions of *dcap-1* with *rfp* and *ife-2* with *gfp* in N2 background | This study |
| BRF118 | N2; *synEx148[P_tiar-1_::gfp::3'UTR^unc-54^ ;rol-6(su1006)]* | Transcriptional fusion of *tiar-1* with *gfp* in N2 background | This study |
| BRF238 | N2; *synEx273[P_tiar-2_::gfp::3'UTR^unc-54^ ;rol-6(su1006)]* | Transcriptional fusion of *tiar-2* with *gfp* in N2 background | This study |
| BRF211 | N2; *synEx264[P_tiar-1_::gfp::tiar-1::3'UTR^tiar-1^ ;rol-6(su1006)]* | Translational fusion of *tiar-1* with *gfp* in N2 background | This study |
| BRF255 | N2; *synEx295[P_tiar-2_::gfp::tiar-2::3'UTR^tiar-2^ ;rol-6(su1006)]* | Translational fusion of *tiar-2* with *gfp* in N2 background | This study |
| BRF120 | N2; *synEx144[P_tiar-3_::tiar-3::gfp::3'UTR^unc-54^ ;rol-6(su1006)]* | Translational fusion of *tiar-3* with *gfp* in N2 background | This study |
| BRF310 | N2; *synEx307[P_myo-3_::gfp::tiar-2::3'UTR^tiar-2^ ;rol-6(su1006)]* | Translational fusion of *tiar-2* with *gfp* under the promoter of *myo-3* muscle-specific gene in N2 | This study |
| BRF312 | N2; *synEx303[P_tiar-1::_rfp::tiar-1::3'UTR^tiar-1^;P_ife-2_::ife-2::gfp::3'UTR^unc-54^  ;rol-6(su1006)]* | Translational fusions of *tiar-1* with *rfp* and *ife-2* with *gfp* in N2 | This study |
| BRF328 | N2; *synEx304[P_tiar-1::_rfp::tiar-1::3'UTR^tiar-1^; P_dcap-1_::dcap-1::gfp::3'UTR^dcap-1^ ;rol-6(su1006)]* | Translational fusions of *tiar-1* with *rfp* and *dcap-1* with *gfp* in N2 background | This study |
| BRF361 | N2; *synEx305[P_tiar-1::_rfp::tiar-1::3'UTR^tiar-1^; P_tiar-2_::gfp::tiar-2::3'UTR^tiar-2^ ;rol-6(su1006)]* | Translational fusions of *tiar-1* with *rfp* and *tiar-2* with *gfp* in N2 background | This study |
| BRF369 | N2; *synEx320[P_dcap-1_::dcap-1::tagrfp::3'UTR^unc-54^ ; P_tiar-2_::gfp::tiar-2::3'UTR^tiar-2^ ;rol-6(su1006)]* | Translational fusions of *dcap-1* with *rfp* and *tiar-2* with *gfp* in N2 background | This study |
| BRF259 | *dcap-1(tm3163); synEx190* | Translational fusion of *dcap-1* with *gfp* in *dcap-1(tm3163)* | This study |
| BRF260 | *tiar-1(tm361); synEx264* | Translational fusion of *tiar-1* with *gfp* in *tiar-1(tm361)* | This study |
| BRF345 | N2; *synEx31[rol-6(su1006)]* | pRF4 plasmid in N2 background | This study |
| BRF354 | *dcap-1(tm3163)*; s*ynEx31* | pRF4 plasmid in *dcap-1(tm3163)* | This study |
| BRF341 | *glp-1(* *e2141); synEx31* | pRF4 plasmid in *glp-1(* *e2141)* | This study |
| BRF294 | *gcn-2(ok871); synEx264* | From crossing *gcn-2(ok871)* males with BRF211 hermaphrodites | This study |
| BRF339 | *gcn-2(ok871); synEx307* | From crossing *gcn-2(ok871)* males with BRF310 hermaphrodites | This study |
| BRF351 | *pek-1(ok275); synEx264* | From crossing males of *pek-1(ok275)* with BRF211 hermaphrodites | This study |
| BRF220 | *ife-2(ok306); synEx190* | Translational fusion of *dcap-1* with *gfp* in *ife-2(ok306)* | This study |
