# Supplementary material for: Diverse Functions of mRNA Metabolism Factors in Stress Defense and Aging of Caenorhabditis elegans
Source: PLoS One. 2014 Jul 25;9(7):e103365. doi: 10.1371/journal.pone.0103365 (PMC4111499; doi:10.1371/journal.pone.0103365)
Supplement: Table S2 — Primers used in this study. (DOCX) [file pone.0103365.s010.docx]

**Table S2:** Primers used in this study

| **Primer** | **Sequence (5' to 3')** | **Used for** |
| --- | --- | --- |
| DCAP-1/1 | CTCCAGAAAATCGACATCGC | *dcap-1(tm3163)* genotyping |
| DCAP-1/2 | GACGCCGATTGAGTGCAC |  |
| DCAP-2/1 | CACGAATTCCGAATACCCC | *dcap-2(RNAi)* construct |
| DCAP-2/2 | CCGCTCGAGTAACGAGACCAAGTACCG |  |
| DCAP-2/3 | GAGCCGCCATCAAGTGTAC | *dcap-2(ok2023)* genotyping |
| DCAP-2/4 | GTACCAATGAGCAAGTTCAAGATG |  |
| DCAP-2/5 | CGTCGAAGGGATCTTCCAG |  |
| TIAR-1/1 | CGGGATCCCTCGAGTCCTCCTTCAACCCACCAG | *tiar-1(RNAi*) construct |
| TIAR-1/2 | CGGGATCCCTCGAGTTATTGATGTCCTCCAGAG |  |
| TIAR-1/3 | CGGGATCCATGTCCTTCTTCAACCC | *tiar-1(tm361)* genotyping |
| TIAR-1/4 | AGCACGCTCAGCTTCTTCTC |  |
| TIAR-2/1 | CGGAAGAAATCGTTTACCCC | *tiar-2(RNAi)* construct |
| TIAR-2/2 | GCTCGAGTCAATTTCCTCCAGTAGATGAAG |  |
| TIAR-2/3 | CCGAGATCTATGGCCACTTCGTTCTACAC | *tiar-2(tm2923)* genotyping |
| TIAR-2/4 | CTCGAATGCCCACGTTAC |  |
| TIAR-3/1 | GTCTGAATTCACAGGTTAACTG | *tiar-3(ok144)* genotyping and RNAi construct^a^ |
| TIAR-3/2 | GCGGATCCCCATTGTGGGAAGGAACTG |  |
| TIAR-3/3 | GAAAGCCGATAACACGTCAG |  |
| GCN-2/1 | GCGATTGATGTTGTTCCAG | *gcn-2(ok871)* genotyping |
| GCN-2/2 | GAGACCACATCCATCGC |  |
| GCN-2/3 | GTGAGTAGACTCGTCCG |  |
| PEK-1/1 | CCGATTGACATTGGAGGAAC | *pek-1(ok275)* genotyping |
| PEK-1/2 | GACACCGAAGTAGATCTCAAG |  |
| PEK-1/3 | TGGAGATACGAGGATGCACC |  |
| PEK-1/4 | CAATCCAAGCTTGCTTTCGG |  |
| PATR-1/1 | CATGCCATGGATTCTAAACTCGGACTGC | *patr-1(RNAi)* construct |
| PATR-1/2 | GCTCTAGATGACTGAATTGTGGACC |  |
| XRN-1/1 | GGTCTAGACCTCTCAAATCTCGACGC | *xrn-1(RNAi)* construct |
| XRN-1/2 | CCGAAGCTTGACCTTCAACACCATCGAC |  |
| LSM-1/1 | GGAGATCTGACTTGCCCGATCCCTATTTAC | *lsm-1(RNAi)* construct |
| LSM-1/2 | GGACTAGTGAAGTTGAAAGCAATCCTCCG |  |
| CGH-1/1 | GCCCCGGGCGAAGGGAGTCGAGTTTGAG | *cgh-1(RNAi)* construct |
| CGH-1/2 | GCCCCGGGTGAAGGTAAGTCTCGGCGTT |  |
| IFE-2/1 | CGATATCGAAAGTCTGTGCGG | *ife-2(ok306)* genotyping |
| IFE-2/2 | AGTGGCTGGTGTGGCAGGA |  |
| IFE-2/3 | ACGGGTGTTTCCAGTCTGA |  |
| EAT-2/1 | GCTAGTCGATTTTCATCATCG | *eat-2(ad465)* genotyping |
| EAT-2/2 | GGCTAACCTTCAAATAGCAAAC |  |

a: primers TIAR-3/1 and TIAR-3/2 were used for both genotyping and RNAi construct.
