# Supplementary material for: Diverse Functions of mRNA Metabolism Factors in Stress Defense and Aging of Caenorhabditis elegans
Source: PLoS One. 2014 Jul 25;9(7):e103365. doi: 10.1371/journal.pone.0103365 (PMC4111499; doi:10.1371/journal.pone.0103365)
Supplement: Table S3 — RNAi plasmids used in this study. (DOCX) [file pone.0103365.s011.docx]

**Table S3:** RNAi plasmids used in this study

| **Construct** | **Description** |
| --- | --- |
| *dcap-1(RNAi)* | *XhoI/EcoRI* digest fragment of FLAG::*dcap-1* (Ding *et al*., 2005) |
| *dcap-2(RNAi)* | *EcoRI/XhoI* digest of PCR with primers DCAP-2/1 and DCAP-2/2 |
| *dcap-1/dcap-2 (RNAi)* | *XhoI* digest fragment of FLAG::*dcap-1* (Ding *et al*., 2005) cloned in *dcap-2* RNAi vector |
| *patr-1(RNAi)* | *NcoI/XbaI* digest of PCR with primers PATR-1/1 and PATR-1/2 |
| *ife-2(RNAi)* | Described in Syntichaki *et al*., 2007 |
| *rsks-1(RNAi)* | Described in Syntichaki *et al*., 2007 |
| *eIF2a(RNAi)* | Described in Rousakis *et al*., 2013 |
| *eIF2Bγ(RNAi)* | *XhoI/XbaI* digest fragment of *ppp-1* |
| *xrn-1(RNAi)* | *XbaI/HindIII* digest of PCR with primers XRN-1/1 and XRN-1/2 |
| *tiar-1(RNAi)* | *XhoI/HindIII* digest of PCR with primers TIAR-1/1 and TIAR-1/2 from TIAR-1-pDONR |
| *tiar-2(RNAi)* | *XbaI/XhoI* digest of PCR with primers TIAR-2/1 and TIAR-2/2 |
| *tiar-3(RNAi)* | *XbaI/NcoI* digest of PCR with primers TIAR-3/1 and TIAR-3/2 |
| *lsm-1(RNAi)* | *BglII/SpeI* digest of PCR with primers LSM-1/1 and LSM1/2 |
| *ain-1(RNAi)* | *EcoRV/BamHI* digest fragment of *ain-1::gfp* (Ding *et al*., 2005) |
| *cgh-1(RNAi)* | *SmaI* digest of PCR with primers CGH-1/1 and CGH-1/2 (cloned in *EcoRV* sites) |

All fragments were cloned into pL4440 (Timmons and Fire, 1998).

All PCR reactions were performed with genomic DNA as template unless otherwise mentioned.
