# Supplementary material for: Diverse Functions of mRNA Metabolism Factors in Stress Defense and Aging of Caenorhabditis elegans
Source: PLoS One. 2014 Jul 25;9(7):e103365. doi: 10.1371/journal.pone.0103365 (PMC4111499; doi:10.1371/journal.pone.0103365)
Supplement: Table S4 — Quantification of number of granules. (DOCX) [file pone.0103365.s012.docx]

**Table S4:** Quantification of number of granules

|  | **Strain** | **Treatment** | **Formed granules**  **(Mean±SD)** | **p-value^d^** |
| --- | --- | --- | --- | --- |
| **Figure**  **1A** | BRF155^a^ | -HS | 11±2.3 |  |
|  |  | +HS | 168±14.9 | <0.0001(***) |
|  | BRF261^a^ | -HS | 27±5.9 |  |
|  |  | +HS | 168±11.5 | <0.0001(***) |
|  | BRF219^a^ | -HS | 11±2.7 |  |
|  |  | +HS | 97±12.3 | <0.0001(***) |
| **Figure**  **2A** | BRF155^a^ | -HS | 2±0.9 |  |
|  |  | +HS | 86±9.5 | <0.0001(***) |
|  |  | Recovery | 3±1.2 | 0.7930(ns) |
|  | BRF261^a^ | -HS | 22±8.6 |  |
|  |  | +HS | 140±14.2 | <0.0001(***) |
|  |  | Recovery | 41±23.3 | 0.4353(ns) |
| **Figure**  **2B** | BRF155^a^ | Control(RNAi) -HS | 0.3±0.3 |  |
|  |  | Control (RNAi) +HS | 98±8.9 | <0.0001(***) |
|  |  | *cgh-1*(RNAi) -HS | 0.6±0.2 |  |
|  |  | *cgh-1*(RNAi) +HS | 5±1.4 | 0.0196(*) |
| **Figure**  **3A** | BRF155^b^ | 1-day adults | 5±2.5 |  |
|  |  | 5-day adults | 101±11.8 | <0.0001(***) |
|  | BRF261^b^ | 1-day adults | 29±5.6 |  |
|  |  | 5-day adults | 128±8.9 | <0.0001(***) |
| **Fig.**  **4A** | BRF155^a^ | Control(RNAi) | 5±1 |  |
|  |  | *xrn-1*(RNAi) | 92±7.9 | <0.0001(***) |
| **Figure**  **6D** | BRF211^b^ | Control | 10±1.4 |  |
|  |  | HS | 78±6.5 | <0.0001(***) |
|  |  | SA | 96±9.0 | <0.0001(***) |
|  | BRF255^c^ | Control | 2±0.5 |  |
|  |  | HS | 38±1.5 | <0.0001(***) |
|  | BRF310^a^ | Control | 5±2.7 |  |
|  |  | HS | 116±13.7 | <0.0001(***) |
|  |  | SA | 63±9.1 | <0.0001(***) |
| **Figure**  **7** | BRF211^b^ | -HS | 9±1.9 |  |
|  |  | +HS 45 min | 49±10.5 | 0.0018(**) |
|  |  | +HS 1.5 h | 72±8.6 | <0.0001(***) |
|  |  | +HS 2.5 h | 94±5.1 | <0.0001(***) |
|  |  | Recovery | 14±1.4 | 0.0578(ns) |
|  | BRF255^c^ | -HS | 2±0.6 |  |
|  |  | +HS 45 min | 9±3.9 | 0.0872(ns) |
|  |  | +HS 1.5 h | 21±3.8 | 0.0019(**) |
|  |  | +HS 2.5 h | 38±4.3 | 0.0002(***) |
|  |  | Recovery | 6±3.1 | 0.1524(ns) |
|  | BRF310^a^ | -HS | 16±5.1 | <0.0001(***) |
|  |  | +HS 45 min | 68±5.5 | <0.0001(***) |
|  |  | +HS 1.5 h | 156±17.3 | <0.0001(***) |
|  |  | +HS 2.5 h | 160±21.1 |  |
|  |  | Recovery | 14±4.7 | 0.7798(ns) |
| **Figure**  **S3A** | BRF70^a^ | Control(RNAi) -HS | 0±0 |  |
|  |  | Control(RNAi) +HS | 62±5.9 | <0.0001(***) |
|  |  | *cgh-1*(RNAi) -HS | 0±0 |  |
|  |  | *cgh-1*(RNAi) +HS | 53±1.7 | <0.0001(***) |
| **Figure**  **S4** | BRF155^b^ | 1-day adults | 16±2.5 |  |
|  |  | 5-day adults | 30±5.8 | 0,0255(*) |
|  |  | 10-day adults | 102±15.7 | <0.0001(***) |
|  |  | 15-day adults | 106±9.5 | <0.0001(***) |

a: number of granules per 2500 μm^2^ per worm

b: number of granules per head

c: number of granules per 100 μm length of excretory cell

d: p-value from log-rank test comparing the number of granules formed under each condition with the respective control. Definitions of used symbols, according to GraphPad Prism 5: ns indicates not significant (p>0.05); * indicates significant (p-value 0.01 to 0.05); ** indicates very significant (p-value 0.001 to 0.01); *** indicates extremely significant (p<0.001).
