# Supplementary material for: Diverse Functions of mRNA Metabolism Factors in Stress Defense and Aging of Caenorhabditis elegans
Source: PLoS One. 2014 Jul 25;9(7):e103365. doi: 10.1371/journal.pone.0103365 (PMC4111499; doi:10.1371/journal.pone.0103365)
Supplement: Table S5 — Lifespan assays in OP-50 plates. (DOCX) [file pone.0103365.s013.docx]

**Table S5:** Lifespan assays in OP-50 plates^a^

| **Strain** | **Treatment** | **Median/Max^b^** | **Mean±s.e.m.^c^** | **n (T/C)^d^** | **p-value^e^** |
| --- | --- | --- | --- | --- | --- |
| N2 (Fig. 5A) | 20°C | 22/31.2 | 21.88±1.25 | 123/11 |  |
| *dcap-1(tm3163)* | >> | 17/26.7 | 17.5±0.5 | 111/20 | <0.0001(***) |
| *dcap-2(ok2023)* | >> | 13/20.8 | 13.5±0.5 | 77/21 | <0.0001 (***) |
| N2 | 20°C | 21/30.9 | 22.83±1.74 | 80/7 |  |
| *dcap-1(tm3163)* | >> | 15/25 | 15±0.577 | 61/16 | <0.0001 (***) |
| *dcap-2(ok2023)* | >> | 11/15.2 | 10.67±0.33 | 100/58 | <0.0001 (***) |
| N2 | 25°C | 11/18.8 | 11.25±0.25 | 137/24 |  |
| *dcap-1(tm3163)* | >> | 9/12.5 | 9.5±0.29 | 127/19 | <0.0001 (***) |
| *dcap-2(ok2023)* | >> | 8/9.6 | 7.67±0.67 | 104/60 | <0.0001 (***) |
| *N2;synEx31* ^f^ | 25°C | 12/19.7 | 12.75±0.75 | 89/2 |  |
| *dcap-1(tm3163); synEx31* ^f^ | >> | 8/9.6 | 7.67±0.67 | 104/60 | <0.0001 (***) |
| *dcap-1(tm3163); synEx190* ^f^ | >> | 11/19.3 | 11.38±0.24 | 88/3 | 0.9437 (ns) |
| *glp-1(e2141)* | 20°C ^g^ | 24/38.1 | 24±0.41 | 135/12 |  |
| *glp-1(e2141); dcap-1(tm3163)* | >> | 18/29.4 | 17.5±1.26 | 124/17 | <0.0001 (***) |
| *glp-1(e2141); dcap-2(ok2023)* | >> | 14/27.4 | 13.5±0.65 | 163/7 | <0.0001 (***) |
| *glp-1(e2141)* | 20°C ^g^ | 23/32.2 | 23.25±0.48 | 93/6 |  |
| *glp-1(e2141); dcap-1(tm3163)* | >> | 18 | 18±0.41 | 111/18 | <0.0001 (***) |
| *dcap-1(tm3163)* | >> | 17 | 17.38±0.24 | 110/10 | <0.0001 (***) |
| N2 | 20°C ^g^ | 22/31.8 | 21.88±0.12 | 114/14 |  |
| *dcap-1(tm3163)* | >> | 17/27.8 | 17.38±0.24 | 110/10 | <0.0001 (***) |
| *glp-1(e2141)* | >> | 23/32.2 | 23.25±0.48 | 93/6 | 0.3917 (ns) |
| *glp-1(e2141); dcap-1(tm3163)* | >> | 18/27.5 | 18±0.41 | 134/38 | <0.0001 (***) |
| *daf-2(e1368)* (Fig. 5D) | 25°C | 43/56.1 | 42.67±0.33 | 97/22 |  |
| *daf-2(e1368); dcap-1(tm3163)* | >> | 35/46.8 | 34±1.73 | 100/8 | <0.0001 (***) |
| *daf-2(e1368); dcap-2(ok2023)* | >> | 23/38.9 | 22.83±2.12 | 103/5 | <0.0001 (***) |
| *eat-2(ad465)* | 25°C | 19/24.2 | 18.33±0.66 | 103/7 |  |
| *eat-2(ad465); dcap-1(tm3163)* | >> | 11/17.3 | 11.17±0.16 | 105/32 | <0.0001 (***) |
| *eat-2(ad465); dcap-2(ok2023)* | >> | 8/12.7 | 7.75±0.48 | 86/19 | <0.0001 (***) |
| *eat-2(ad465)* | 25°C | 20/25.3 | 19.8±0.37 | 183/19 |  |
| *eat-2(ad465); dcap-1(tm3163)* | >> | 13/19.1 | 12.75±0.47 | 116/33 | <0.0001 (***) |
| N2 | 25°C | 11/18.8 | 11.25±0.25 | 137/24 |  |
| *ife-2(ok306)* | >> | 13/20.9 | 13.5±2.88 | 148/1 | <0.0001 (***) |
| *ife-2(ok306); dcap-1(tm3163)* | >> | 10/13.6 | 9.75±0.48 | 122/14 | <0.0001 (***) |
| N2 | 20°C | 19/30.1 | 19.75±0.48 | 90/7 |  |
| *tiar-1(tm361)* | >> | 12/21.5 | 12.25±0.14 | 106/8 | <0.0001 (***) |
| *tiar-2 (tm2923)* | >> | 18/28.6 | 18.63±0.24 | 101/12 | 0.1259 (ns) |
| *tiar-3 (ok144)* | >> | 18/22.3 | 17.5±0.5 | 60/9 | 0.0008 (**) |
| N2 (Fig. 8B) | 20°C | 21/27.9 | 20.83±0.17 | 149/13 |  |
| *tiar-1(tm361)* | >> | 14/22.5 | 15±0.58 | 128/22 | <0.0001 (***) |
| *tiar-2 (tm2923)* | >> | 19/29.6 | 19±0.58 | 146/2 | 0.0150 (*) |
| *tiar-3 (ok144)* | >> | 20/27 | 20.33±0.33 | 147/7 | 0.0028 (**) |
| *tiar-1(tm361); tiar-2(tm2923)* | >> | 10/14.6 | 9.75±0.25 | 120/25 | <0.0001 (***) |
| N2 | 20°C | 22/31.9 | 22±0.57 | 92/9 |  |
| *tiar-1(tm361)* | >> | 13/17.6 | 13.33±0.33 | 79/23 | <0.0001 (***) |
| *tiar-1(tm361); synEx264* ^f^ | >> | 22/29.4 | 21.67±0.33 | 61/10 | 0.7317 (ns) |
| N2 | 25°C | 13/18.3 | 13±0.57 | 83/9 |  |
| *tiar-1(tm361)* | >> | 9/14.2 | 9.12±0.12 | 97/3 | <0.0001 (***) |
| *tiar-2 (tm2923)* | >> | 10/17.2 | 9.5±0.28 | 102/14 | 0.0003 (**) |
| *tiar-3 (ok144)* | >> | 13/17.3 | 12.5±0.29 | 79/1 | 0.5786 (ns) |
| *tiar-1(tm361); tiar-2(tm2923)* | >> | 8/11.3 | 7.67±0.33 | 72/15 | <0.0001 (***) |
| *glp-1(e2141)* | 20°C ^g^ | 25/56 | 24.5±1.5 | 112/5 |  |
| *glp-1(e2141); tiar-1(tm361)* | >> | 19/33 | 18.5±0.5 | 253/1 | <0.0001 (***) |
| *glp-1(e2141); tiar-2(tm2923)* | >> | 14/52.5 | 14±0.58 | 217/3 | <0.0001 (***) |
| *glp-1(e2141); tiar-3(ok144)* | >> | 28/56.3 | 27.75±0.25 | 219/12 | 0.9048 (ns) |
| *glp-1(e2141)* | 20°C ^g^ | 23/61 | 22.5±0.5 | 108/8 |  |
| *glp-1(e2141); tiar-1(tm361)* | >> | 18/32.8 | 18.25±0.25 | 248/2 | <0.0001 (***) |
| *glp-1(e2141); tiar-2(tm2923)* | >> | 13/40.9 | 13.17±0.44 | 137/2 | <0.0001 (***) |
| *glp-1(e2141); tiar-3(ok144)* | >> | 28/46.9 | 27.33±0.89 | 137/4 | 0.0258 (*) |

a: Independent repeats of each lifespan experiment. Data set within each panel were done in parallel and statistical analysis was performed within the data set.

b: Max lifespan is the mean of the last 10% surviving worms (days).

c: Mean lifespan and standard error of the mean (s.e.m.) of 2-4 plates (days).

d: Total number (T) of worms/censored animals (C).

e: p-value from log-rank test comparing a mutant strain to N2 or a double mutant to the single one. Definitions of used symbols, according to GraphPad Prism 5: ns indicates not significant (p>0.05); * indicates significant (p-value 0.01 to 0.05); ** indicates very significant (p-value 0.001 to 0.01); *** indicates extremely significant (p<0.001).

f: See Table S1.

g: Eggs were laid at 25°C and the germ-line deficient young adults were transferred to 20°C, for the rest of their life.
