# Supplementary material for: Diverse Functions of mRNA Metabolism Factors in Stress Defense and Aging of Caenorhabditis elegans
Source: PLoS One. 2014 Jul 25;9(7):e103365. doi: 10.1371/journal.pone.0103365 (PMC4111499; doi:10.1371/journal.pone.0103365)
Supplement: Table S6 — Lifespan assays in RNAi plates. (DOCX) [file pone.0103365.s014.docx]

**Table S6**: Lifespan assays in RNAi plates^a^

| **Genotype and RNAi** | **Treatment** | **Median/Max^b^** | **Mean±s.e.m.^c^** | **n (T/C)^d^** | **p-value^e^** |
| --- | --- | --- | --- | --- | --- |
| N2/control*(RNAi)* (eggs) | 20°C | 22/31.8 | 21.67±0.33 | 83/3 |  |
| N2/*dcap-*1*(RNAi)* >> | >> | 17/23.9 | 17.67±0.88 | 99/10 | <0.0001 (***) |
| N2/*dcap-1/2(RNAi)* >> | >> | 18/22.9 | 17.75±0.75 | 95/5 | <0.0001 (***) |
| N2/control*(RNAi)* | 20°C | 21/31.5 | 21.67±0.33 | 97/4 |  |
| N2/*dcap-*1*(RNAi)* | >> | 20/29.1 | 20.00±0.58 | 90/4 | 0.0196 (*) |
| N2/*dcap-2(RNAi)* | >> | 19/30.2 | 19.67±0.67 | 94/5 | 0.0171 (*) |
| N2/*dcap-1/2 (RNAi)* | >> | 19/29 | 18.67±0.33 | 101/7 | 0.0008 (***) |
| N2/control*(RNAi)* | 20°C | 22/31.8 | 21.33±0.33 | 169/7 |  |
| N2/*dcap-1(RNAi)* | >> | 21/30.5 | 20.75±0.48 | 167/3 | 0.7164 (ns) |
| N2/*dcap-2(RNAi)* | >> | 20/30.2 | 20.67±0.33 | 124/2 | 0.4228 (ns) |
| N2/*dcap-1/2* RNAi | >> | 19/30.1 | 19.67±0.33 | 126/3 | 0.0244 (*) |
| N2*/part-1(RNAi)* | >> | 18/28.7 | 18.25±0.75 | 97/2 | 0.0032 (**) |
| N2/*xrn-1(RNAi)* | >> | 17/28.3 | 17.33±0.67 | 90/5 | 0.0018 (**) |
| N2/control*(RNAi)* (Fig. 5B) | 20°C | 22/31.7 | 22±0.61 | 70/0 |  |
| N2/*dcap-1/2(RNAi)* | >> | 21/26.3 | 21±0.0 | 76/1 | 0.0060 (**) |
| N2/*part-1(RNAi)* | >> | 19/26.2 | 18±1.16 | 72/0 | 0.0002 (***) |
| N2/*xrn-1(RNAi)* | >> | 17/27.9 | 17.33±0.67 | 94/7 | 0.0005 (***) |
| N2/control*(RNAi)* | 20°C | 21/ 30 | 20.67 ±0.67 | 87/9 |  |
| N2/*cgh-1(RNAi)* | >> | 19/29.3 | 18.67±0.67 | 87/4 | 0.0388 (*) |
| N2/control*(RNAi)* | 25°C | 14/20.6 | 14±1 | 69/3 |  |
| N2/*dcap-1(RNAi)* | >> | 15/22.2 | 14.75±1.25 | 76/1 | 0.4070 (ns) |
| N2/*dcap-2(RNAi)* | >> | 15/21.2 | 15±0 | 70/0 | 0.3654 (ns) |
| N2/*dcap-1/2(RNAi)* | >> | 13/19.3 | 12.5±1.5 | 73/2 | 0.0464 (*) |
| *daf-2(e1370)*/control*(RNAi)* | 20°C | 49/62.6 | 49.5±1.5 | 86/8 |  |
| *daf-2(e1370)/dcap-1(RNAi)* | >> | 46/58.5 | 46±0.58 | 119/3 | 0.0007 (***) |
| *daf-2(e1370/ dcap-2(RNAi)* | >> | 44/59.9 | 43.67±1.45 | 123/3 | 0.0032 (**) |
| *daf-2(e1370)/dcap-1/2(RNAi)* | >> | 44/56.3 | 43.75±1.25 | 72/5 | 0.0002 (***) |
| *ife-2(ok306)/*control*(RNAi)* | 20°C | 26/38.1 | 25.75±0.75 | 88/0 |  |
| *ife-2(ok306)*/*dcap-1/2i(RNAi)* | >> | 21/31.4 | 21±0 | 99/6 | <0.0001 (***) |
| *ife-2(ok306)*/*patr-1(RNAi)* | >> | 23/32.8 | 23±2 | 88/5 | 0.0077 (**) |
| *ife-2(ok306)*/control*(RNAi)* | 20°C | 25/38.1 | 25.33±0.33 | 111/5 |  |
| *ife-2(ok306)/dcap-1/2(RNAi)* | >> | 21/31.4 | 21.33±0.6 | 116/1 | <0.0001 (***) |
| *rsks-1(ok1255)*/control*(RNAi)* | 20°C | 27/37 | 26.75±0.63 | 98/35 |  |
| *rsks-1(ok1255)*/*dcap-1/2* RNAi | >> | 24/32.6 | 23.75±0.48 | 102/18 | 0.0003 (***) |
| *rsks-1(ok1255)*/*patr-1* RNAi | >> | 23/31.4 | 22.75±0.85 | 99/29 | <0.0001 (***) |
| *rsks-1(ok1255)*/control*(RNAi)* | 20°C | 25/31 | 25±0.0 | 57/8 |  |
| *rsks-1(ok1255)*/*dcap-1(RNAi)* | >> | 21/26.4 | 21±0.0 | 67/22 | 0.0031 (**) |
| *rsks-1(ok1255)*/*dcap-2(RNAi)* | >> | 20/27 | 21±1 | 70/20 | 0.0037 (**) |
| *rsks-1(ok1255)*/*dcap-1/2(RNAi)* | >> | 21/26.5 | 21.5±1 | 69/28 | 0.0039 (**) |
| *rsks-1(ok1255)*/*patr-1(RNAi)* | >> | 22/28 | 21±0.0 | 62/21 | 0.0047 (**) |
| *glp-1(e2141)*/control*(RNAi)* | 20°C ^g^ | 27/37.4 | 22.67±0.89 | 120/3 |  |
| *glp-1(e2141)*/*dcap-1(RNAi)* | >> | 23/31.4 | 23±0.00 | 120/6 | <0.0001 (***) |
| *glp-1(e2141)*/*dcap-2(RNAi)* | >> | 23/32.9 | 23.5±0.76 | 117/4 | 0.0003 (***) |
| *glp-1(e2141)*/*dcap-1/2(RNAi)* | >> | 22/31.8 | 22±0.57 | 121/1 | <0.0001 (***) |
| *glp-1(e2141)*/*xrn-1(RNAi)* | >> | 23/ 32.8 | 22.33±0.67 | 116/2 | <0.0001 (***) |
| *glp-1(e2141)*/control*(RNAi)* | 20°C ^g^ | 23/37.3 | 23.33±0.33 | 154/12 |  |
| *glp-1(e2141)*/*dcap-1/2(RNAi)* | >> | 23/34.3 | 23±0.58 | 148/5 | 0.0245 (*) |
| *glp-1(e2141)*/*xrn-1(RNAi)* | >> | 21/ 30.7 | 21.33±0.67 | 146/15 | <0.0001 (***) |
| N2/control*(RNAi)* (Fig. 8B) | 20°C | 22/32.2 | 21.67±0.33 | 151/7 |  |
| N2/*tiar-1(RNAi)* | >> | 16/27 | 16.33±1.45 | 112/7 | <0.0001 (***) |
| N2/*tiar-2(RNAi)* | >> | 20/32 | 20.17±1.3 | 143/8 | 0.1112 (ns) |
| N2*/tiar-3(RNAi)* | >> | 18/24.8 | 19±1 | 72/10 | <0.0001 (***) |
| N2/control*(RNAi)* | 20°C | 22/31.7 | 22±0.61 | 70/0 |  |
| N2/*tiar-1(RNAi)* | >> | 20/27.5 | 20±0.58 | 75/6 | 0.0084 (**) |
| N2/*tiar-2(RNAi)* | >> | 20/31.9 | 20±0.58 | 66/0 | 0.6641 (ns) |
| N2/control*(RNAi)* | 25°C | 11/ 22.4 | 12 ± 1.73 | 140/22 |  |
| N2/*tiar-1(RNAi)* | >> | 9/12.5 | 9.33 ± 0.33 | 103/2 | <0.0001 (***) |
| N2*/tiar-3(RNAi)* | >> | 10/18.1 | 11± 1 | 130/8 | 0.0072 (**) |
| N2/control*(RNAi)* | 25°C | 13/22.8 | 13.75±0.63 | 174/4 |  |
| N2/*tiar-1(RNAi)* | >> | 12/17.3 | 11.75±0.48 | 180/7 | <0.0001 (***) |
| N2/*tiar-2(RNAi)* | >> | 11/17.4 | 10.88±0.31 | 98/0 | <0.0001 (***) |

a: Independent repeats of each lifespan experiment. Data set within each panel were done in parallel and statistical analysis was performed within the data set. RNAi was achieved by feeding animals bacteria expressing dsRNA of the indicated gene(s); “control” indicates bacteria containing empty vector plasmid.

b: Max lifespan is the mean of the last 10% surviving worms (days).

c: Mean lifespan and standard error of the mean (s.e.m.) of 2-4 plates (days).

d: Total number (T) of worms/censored animals (C).

e: p-value from log-rank test comparing an RNAi-treated strain to N2 control (RNAi). Definitions of used symbols, according to GraphPad Prism 5: ns indicates not significant (p>0.05); * indicates significant (p-value 0.01 to 0.05); ** indicates very significant (p-value 0.001 to 0.01); *** indicates extremely significant (p<0.001).

g: Eggs were laid at 25°C and the germline deficient young adults were transferred to 20°C, for the rest of their life.
